# *Citrus* fruits as a treasure trove of active natural metabolites that potentially provide benefits for human health

**DOI:** 10.1186/s13065-015-0145-9

**Published:** 2015-12-24

**Authors:** Xinmiao Lv, Siyu Zhao, Zhangchi Ning, Honglian Zeng, Yisong Shu, Ou Tao, Cheng Xiao, Cheng Lu, Yuanyan Liu

**Affiliations:** School of Chinese Materia Medica, Beijing University of Chinese Medicine, Beijing, 100029 China; Institute of Clinical Medicine, China-Japan Friendship Hospital, Beijing, 100029 China; Institute of Basic Research in Clinical Medicine, China Academy of Chinese Medical Sciences, Beijing, 100700 China; School of Chinese Medicine, Hong Kong Baptist University, Kowloon Tong, Hong Kong SAR, 999077 China

**Keywords:** *Citrus* fruits, Secondary metabolites, Bioactivities, Human health, Flavonoids

## Abstract

*Citrus* fruits, which are cultivated worldwide, have been recognized as some of the most high-consumption fruits in terms of energy, nutrients and health supplements. What is more, a number of these fruits have been used as traditional medicinal herbs to cure diseases in several Asian countries. Numerous studies have focused on *Citrus* secondary metabolites as well as bioactivities and have been intended to develop new chemotherapeutic or complementary medicine in recent decades. *Citrus*-derived secondary metabolites, including flavonoids, alkaloids, limonoids, coumarins, carotenoids, phenolic acids and essential oils, are of vital importance to human health due to their active properties. These characteristics include anti-oxidative, anti-inflammatory, anti-cancer, as well as cardiovascular protective effects, neuroprotective effects, etc. This review summarizes the global distribution and taxonomy, numerous secondary metabolites and bioactivities of *Citrus* fruits to provide a reference for further study. Flavonoids as characteristic bioactive metabolites in *Citrus* fruits are mainly introduced.

**Electronic supplementary material:**

The online version of this article (doi:10.1186/s13065-015-0145-9) contains supplementary material, which is available to authorized users.

## Background

*Citrus* fruits, which belong to the genus *Citrus* of the family Rutaceae, are of various forms and sizes (from round to oblong), commonly known as oranges, mandarins, limes, lemons, grapefruits and citrons. The sensory attributes of fruits (color, sweet taste, bitterness, and astringency) constitute decisive organoleptic and commercial properties [1]. *Citrus* species are consumed mainly as fresh or raw materials for juices or are canned as segments. Additionally, *Citrus* fruits can also be used in the food, beverage, cosmetic and pharmaceutical industries as additives, spices, cosmetic ingredients and chemoprophylactic drugs, respectively [2, 3].

*Citrus* fruits are good sources of nutrition with an ample amount of vitamin C. Besides, the fruits are abundant in other macronutrients, including sugars, dietary fiber, potassium, folate, calcium, thiamin, niacin, vitamin B6, phosphorus, magnesium, copper, riboflavin and pantothenic acid [4]. However, secondary metabolites are an especially popular topic in the present research. These constituents, also known as phytochemicals, are small molecules that are not strictly necessarily for the survival of the plants but represent pharmacological activity. *Citrus* fruits contain a number of secondary metabolites, such as flavonoids, alkaloids, coumarins, limonoids, carotenoids, phenol acids and essential oils. These active secondary metabolites show several bioactivities of vital importance to human health, including anti-oxidative, anti-inflammatory, anti-cancer, as well as cardiovascular protective effects, neuroprotective effects, etc. In addition, *Citrus* fruits have been used as traditional medicinal herbs in several Asian countries, such as China, Japan and Korea. Nine traditional Chinese medicines have been recorded in the Chinese Pharmacopoeia for appropriate medical use from six *Citrus* species [5]: *C.reticulata* Blanco, *C.medica* L. var. sarcodactylis Swingle, *C.medica* L., *C.wilsonii* Tanaka, *Citrusaurantium* L. and *C.sinensis* Osbeck. These peels or whole fruits (mature or immature) are known to treat indigestion, cough, skin inflammation, muscle pain, and ringworm infections, as well as to lower blood pressure.

This review summarizes the global distribution and taxonomy, numerous secondary metabolites and bioactivities related to human health of *Citrus* fruits. Especially, flavonoids as the main characteristic metabolites in *Citrus* fruits, which can provide benefit for human health based on their multiple bioactivities. Then, the secondary metabolites variation among different species and fruit parts were mentioned to provide a better guide for our daily use and related industries.

## Distribution and taxonomy

**Fig. 1:**
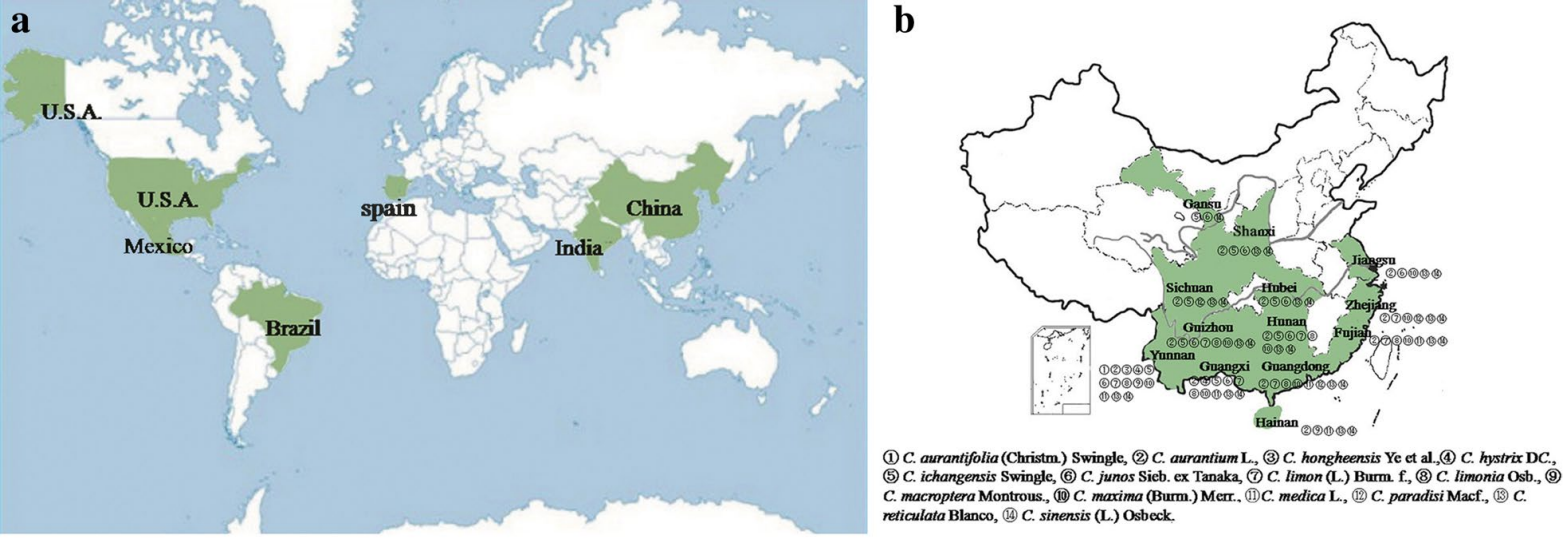
**a** Top six *Citrus* fruits-producing countries in the world. *C*itrus species are grown in 140 countries, though production shows geographical concentration in certain areas. China, Brazil, the USA, India, Mexico, and Spain are the world’s top 6 *C*itrus fruit-producing countries, representing close to two-thirds of global production. China is the first leading country as producers which had produced 32,221,345 tons of *C*itrus fruit in 2012. Brazil is the second production country of *C*itrus fruits with 20,258,507 tons in 2012. And the USA. India, Mexico and Spain also play dominant roles in *C*itrus production which all produced more than 5,000,00 tons in 2012. **b** Distribution of 14 *Citrus*-varieties in the major *Citrus*-producing provinces of China. There are 14 *C*itrus varieties distributed in 13 provinces in China. (*1*) *C.* *aurantifolia* (Christm.) Swingle is mainly distributed in Yunnan province; (*2*) *C.* *aurantium* L. is mainly distributed in Fujian, Guangdong, Guangxi, Guizhou, Hainan, Hubei, Hunan, Jiangsu, Shaanxi, Sichuan, Yunnan, Zhejiang, provinces; (*3*) *C.* *hongheensis* Ye et al. is mainly distributed in Yunnan province; (*4*) *C.* *hystrix* D*C.* is mainly distributed in Guangxi, Yunnan province; (*5*) *C.* *ichangensis* Swingle is mainly distributed in Gansu, Guangxi, Guizhou, Hubei, Hunan, Shaanxi, Sichuan, Yunnan provinces; (*6*) *C.* *junos* Sieb. ex Tanaka is mainly distributed in Gansu, Guangxi, Guizhou, Hubei, Hunan, Jiangsu, Shaanxi, Yunnan provinces; (*7*) *C.* *limon* (L.) Burm. f. is mainly distributed in Fujian, Guangdong, Guangxi, Guizhou, Hunan, Yunnan, Zhejiang provinces; (*8*) *C.* *limonia* Osb. is mainly distributed in Fujian, Guangdong, Guangxi, Guizhou, Hunan, Yunnan provinces; (*9*) *C. macroptera* Montrous. is mainly distributed in Hainan, Yunnan provinces; (*10*) *C.* *maxima* (Burm.) Merr. is mainly distributed in Fujian, Guangdong, Guangxi, Guizhou, Hunan, Jiangsu, Yunnan, Zhejiang provinces; (*11*) *C.* *medica* L. is mainly distributed in Fujian, Guangdong, Guangxi, Hainan, Yunnan provinces; (*12*) *C.* *paradisi* Macf. is mainly distributed in Guangdong, Sichuan, Zhejiang provinces; (*13*) *C.* *reticulata* Blanco is mainly distributed in Fujian, Guangdong, Guangxi, Guizhou, Hainan, Hubei, Hunan, Jiangsu, Shaanxi, Sichuan, Yunnan, Zhejiang provinces; (*14*) *C.* *sinensis* (L.) Osb. is mainly distributed in Fujian, Gansu, Guangdong, Guangxi, Guizhou, Hainan, Hubei, Hunan, Jiangsu, Shaanxi, Sichuan, Yunnan, Zhejiang provinces

According to statistics of FAOSTAT [6], *Citrus* species are grown all over the world in more than 140 countries, with more than 8.7 million hectares and about 131 million tons of fruits produced in 2012. And China, Brazil, the U.S.A., India, Mexico, and Spain are the world’s leading *Citrus* fruit-producing countries (see Fig. 1a), representing close to two-thirds of global production. In China, citriculture has existed traditionally, and the *Citrus* varieties have been naturally selected [7] (see Fig. 1b): (1) *C.aurantifolia* (Christm.) Swingle, (2) *C.aurantium* L., (3) *C.hongheensis* Ye et al., (4) *C.hystrix* D*C.*, (5) *C.ichangensis* Swingle, (6) *C.junos* Sieb. ex Tanaka, (7) *C.limon* (L.) Burm. f., (8) *C.limonia* Osb., (9) *C. macroptera* Montrous., (10) *C.maxima* (Burm.) Merr., (11) *C.medica* L., (12) *C.paradisi* Macf., (13) *C.reticulata* Blanco, (14) *C.sinensis* (L.) Osb.

The genus *Citrus* belongs to the subtribe Citrinae, tribe Citreae, subfamily Aurantioideae of the family Rutaceae. However, continual taxonomic study appears to be very complicated and controversial, mainly due to sexual compatibility between *Citrus* species and related genera, the high frequency of bud mutations, apomixis (e.g., adventitious embryony) [8]. Consequently, there has been no consensus among taxonomists as to the actual number of *Citrus* species. The most widely accepted taxonomic systems for *Citrus* are those of Swingle and Reece [9] and Tanaka [10], who recognized 16 and 162 species, respectively. Later, phylogenetic analysis indicated only three true species within the cultivated *Citrus* [11], i.e., *C.medica* L. (citron), *C.reticulata* Blanco (mandarin) and *C.maxima* (Burm.) Merr. (pummelo). In order to be convenient, the existing taxonomic systems are combined currently.

Because morphological characters are of limited use, studies have mainly focused on new taxonomy methods, i.e., chemotaxonomy. 66 *Citrus* species and near-*Citrus* relatives can be cited in accordance with Tanaka’s classification system with 24 flavonoids [12]. Flavanones were used as chemotaxonomic markers to distinguish 77 *Zhishi* (traditional Chinese medicine) samples from three *Citrus* species [13]. Another study suggested that the content of certain monoterpenes could be as taxonomic markers between *C.sinensis* Osbeck and *C. junos* Sieb. ex Tanaka [14].

## Active secondary metabolites

Plentiful active natural metabolites including flavonoids, alkaloids, coumarins, limonoids, carotenoids, phenolic acids and essential oils, have been found in *Citrus* fruits. Tables in additional files have summarized these secondary metabolites isolated from peel, pulp, seed, pressed oil, juice or whole fruit from 31 common species to give a systematical profile. By these at least, the types of *Citrus*-derived secondary metabolites vary among different *Citrus* species and different fruit parts. Moreover, flavanones, synephrine, auraptene and limonin are the most dominants among the flavonoids, alkaloids, coumarins and limonoids groups, respectively.

In Additional file 1, 48 types of flavonoids from 22 common *Citrus* species of different fruit parts (peel, pulp, seed, pressed oil, juice or whole fruit) have been summarized. These flavonoids belong to the five classes: flavones, flavonols, flavanones, flavanonols and polymethoxylated flavones. Anthocyanins, an uncommon class of flavonoid, only appears in blood oranges of limited data in different fruit parts [15]. Among *Citrus*-derived flavonoids, flavanones comprise approximately 95 % of the total flavonoids [16]. And flavones, flavonols and polymethoxylated flavones present in lower concentration. In addition, some of flavonoids are unique to *Citrus* plants. *Citrus*-derived flavonoids are present in glycoside or aglycone forms, and usually do not occur naturally as aglycones but rather as glycosides, in which the aglycones are linked to a sugar moiety [17]. Among the aglycone forms, naringenin, hesperetin, apigenin, nobiletin, tangeretin and quercetin are widely detected (see Additional file 1). For glycoside forms, O-glycosides, C-glycosides, rutinosides, glucosides and neohesperidosides are common. Naringin (neohesperidoside), neohesperidin (neohesperidoside), narirutin (rutinoside), and hesperidin (rutinoside) are commonly present in major quantities. Sinensetin, isosinensetin, nobiletin, tangeretin, which all belong to polymethoxylated flavones, exist only as aglycones because the binding sites for sugar moieties are not occupied by hydroxyl moieties [18].

In Additional file 2, alkaloids, coumarins, limonoids, carotenoids, phenolic acids and essential oils have also been well summarized from different *Citrus* species and different fruit parts. Active alkaloids are abundant in *C.aurantium* compared to other *Citrus* species, especially synephrine, which comprises more than 85 % of the total protoalkaloid content [19]. Additionally, *N*-methyltyramine has been found at much higher concentrations than octopamine, tyramine or hordenine [20]. Coumarins are commonly found in *Citrus* plants (high concentration in peels). Auraptene (7-geranyloxycoumarin) is a major coumarin in *Citrus* plants. Limonoids are unique compounds occurring in the Meliaceae and Rutaceae family. *Citrus* (a genus in the family Rutaceae) limonoids are highly oxygenated triterpenoids, which are present as aglycones, glucosides, and A-ring lactones. Also, *Citrus* limonoids are the metabolic precursors to limonoid aglycones and glucosides [21]. Limonin and limonin glucoside (see Additional file 2) are the most abundant limonoids for the majority of *Citrus* species. Carotenoids are a large family of isoprenoid compounds that impart yellow, orange, and red pigments to many plants as well as the yellow-to-orange color of *Citrus* fruits. Lutein, zeaxanthin and β-cryptoxanthin, β-carotene, can be found in significant quantities in tangerines and oranges ([22], see Additional file 2). Investigations have shown that the majority of phenolic acids in *Citrus* fruits are present in bound forms [23].

In Additional file 3, Citrus-derived volatile compounds from 15 common *Citrus* Species have been summarized. These compounds are roughly divided into 6 groups: monoterpene hydrocarbons, sesquiterpene hydrocarbons, alcohols, aldehydes, esters & ketones and Oxides. These volatile compounds are mainly come from peels of *Citrus* fruits that have many oil chambers of unique aroma flavors, differ depending on the species and variety.

## Bioactivities

Owing to these metabolites, *Citrus* fruits exhibit plentiful bioactivities including anti-oxidant, anti-inflammatory, anti-cancer, anti-microbial and anti-allergy activities, as well as cardiovascular effect, neuroprotective effect, hepatoprotective effect, obesity control, etc. Note that flavonoids (especially flavanone, flavanonol and methoxylated flavones) are more active compared to other secondary metabolites in *Citrus* for their remarkable various bioactivities. Studies on plentiful bioactivities from hesperetin/hesperidin (flavanone) [24–28], naringenin/naringin (flavanone) [29–34], tangeretin (polymethoxylatedflavone) [35–37] and nobiletin (polymethoxylatedflavone) [36, 38–41] have been widely reported.

### Anti-oxidant

Reactive oxygen species (ROS) are chemically derived from oxygen such as superoxide anion, hydroxyl radicals and hydrogen peroxide in living organisms by amount of metabolism pathways, while anti-oxidant system is able to defend against it to keep balance [42]. However, modern lifestyle involves a number of factors that may raise the level of ROS which play a critical role in the pathogenesis of various diseases such as aging, arthritis, cancer, inflammation, and heart disease, and cause oxidative stress. Citrus extracts such as *Citrus karna* peel extracts, *Citrus* *limetta* peel extracts and *Citrus bergamia* juice extracts were found to have potential antioxidant bioactivity [43–45]. *Citrus* fruits are reported to have a good anti-oxidant ability especially because of their phenolic compounds with poly-hydroxyl groups, including phenolic acids, flavonoids and their derivatives [46]. The primary anti-oxidant mechanisms of phenolic compounds are listed below:

- Direct absorption and neutralization of free radicals [47].
- Inhibition of enzymes associated with ROS pathways: NADPH oxidase, xanthine oxidase and myeloperoxidase [48].
- Enhancement of the activities of human anti-oxidant enzymes: superoxide dismutase, catalase, etc. [49].

#### Flavonoids

The juices from green and ripe chinotto (*C.myrtifolia* Raf.), which were full of flavonoid, was tested by DPPH· radical bleaching and superoxide-anion scavenging, and it was shown that immature chinotto fruits, in particular, yield a juice with a remarkable anti-oxidant power [50]. The anti-oxidant activity of the flavonoid mixture isolated from the *Citrus* peel was determined in terms of the DPPH· and ABTS·^+^ scavenging and the reducing power assay in a concentration range from 25 to 500 mg/L, and its anti-oxidant activity increased in a dose-dependent manner [51]. Sun et al. [52] using FRAP, DPPH, and ABTS assays detected immature fruits drops of nine *Citrus* varieties cultivated in China and determined that the anti-oxidant activity, which varied significantly among the species, was highest in *Citrus poonensis* Hort. ex Tanaka and *Citrus unshiu* Marc. cv Owari and lowest in *Citrus paradise* Macf. Changshanhuyou, *Citrus grandis* (L.) Osbeck cv Foyou, and *Citurs limon* (L.) Burm.f. cv Eureka. Different anti-oxidant assays have applied to evaluate anti-oxidant activity. For instance, quercetagetin showed strong DPPH radical-scavenging activity (IC_50_ 7.89 mol L^−1^) but much lower hydroxyl radical-scavenging activity (IC_50_ 203.82 µmol L^−1^). In vivo, hesperetin was administered orally and acted as a potent antioxidative agent against Cd-induced testicular toxicity in rats [24]. Hesperetin increased the glutathione and glutathione dependent enzymes in the testes of rats, by which it effectively reduced the Cd-induced oxidative stress and restored the activities of ATPases. Aranganathan and Nalini reported that hesperetin exerted an anti-lipoperoxidative effect and thereby restored the membrane-bound ATPase activity in Cd-intoxicated rat testes [53].

#### Phenolic acids

There were positive correlations among the results of the anti-oxidant capacities and total phenolic acids contents of the Tarhana samples [54]. The anti-oxidant potency composite index showed wide variations, ranging from 58.84 to 98.89 in the 14 studied wild mandarin genotypes native to China, due to different phenolic compounds’ levels, including phenolic acids. Ogiwara et al. [55] found that caffeic, chlorogenic, and ferulic acids scavenged various radicals, such as superoxide anions and hydroxy radicals. Citric acids from *Citrus* have been found to show anti-oxidant activity in lipopolysaccharide (LPS)-treated mice [56]. Korani et al. [57] demonstrated that gallic acid has a beneficial activity against 2VO-induced cognitive deficits via enhancement of the cerebral anti-oxidant defense. Among the phenolic acid group, gallic acid with three hydroxyl groups on the aromatic ring was the strongest anti-oxidant [58]. In contrast, the monosubstituted phenolic acids (*p*-coumaric acid, *o*-coumaric acid, and 4-OH-phenylacetic acid) showed very low activity. In addition, the radical-scavenging activities of phenolic acids are related to their hydroxyl group characteristics in the order: gallic > gentisic > syringic > caffeic > protocatechuic > sinapic > ferulic > isoferulic > vanillic > p-coumaric > o-coumaric > m-coumaric > salicylic ≫ p-hydroxybenzoic [59].

#### Essential oils

Singh et al. [60] reported that monoterpenic essential oils were natural anti-oxidants. Choi et al. [61] found that the radical-scavenging activity of 34 types of *Citrus* essential oils on DPPH ranged from 17.7 to 64 %. These activities were determined to be higher when the oils contained geraniol, terpinolene and γ-terpinene. However, the bioactivity of the essential oils generally resulted from a complex interaction between its constituents, which produced both synergistic and antagonistic responses [62].

#### Coumarins

The accumulating data from studies revealed that dihydroxycoumarins were better anti-oxidants than monohydroxycoumarins and that the OH groups positioned near C6 and C7 in the coumarin skeleton played an important role in the inhibition of mushroom tyrosinase [63].

### Anti-inflammatory

Inflammation is a very complex response that is mediated by inflammatory cytokines including tumor necrosis factor-alpha (TNF-α), interleukin-1β and interleukin-6 as well as a cascade of molecular mediators including inducible nitric oxide synthase (iNOS), cyclooxygenase-2 (COX-2), which are all closely regulated by the organism. And these inflammatory cytokines are active in the pathogenesis of various chronic inflammatory diseases such as multiple sclerosis, Parkinson’s disease, Alzheimer’s disease and colon cancer [64]. Orange (*C. aurantium* L.) peel extract was found to suppress UVB-induced COX-2 expression and PGE2 production in HaCaT cells, and acted as a peroxisome proliferator-activated receptor (PPAR)-c agonist [65]. Flavonoids, coumarin and volatile oil from *Citrus* fruit are showing anti-inflammatory activity, which can be used as supplement to protect against or ameliorate this chronic inflammatory diseases.

#### Flavonoids

Naringin reduced lipopolysaccharide- or infection-induced endotoxin shock in mice, attenuated chronic pulmonary neutrophilic inflammation in cigarette smoke-exposed rats [29]. And its aglycone, naringenin, exerted anti-inflammatory activities in macrophages and in human blood [66]. Hesperidin exerted noticeable in vivo anti-inflammatory systemic effects in mouse models of LPS-induced lung inflammation and of endotoxin-induced infection [25], in rat models of rheumatoid arthritis and against inflammation in mouse skin [26]. Nobiletin dose-dependently reduced the nitric oxide (NO) levels and decreased iNOS expression at the protein, mRNA and antisense transcript levels [38]. Sudachitin had been found to inhibit NO production by suppressing the expression of iNOs in LPS-stimulated macrophages, to exhibit anti-inflammatory activity, and was a more potent anti-inflammatory agent than nobiletin [67]. In addition, quercetin was known to possess strong anti-inflammatory capacities [68]. Data suggested that flavone suppresses iNOS expression via a mechanism that was similar to that of nobiletin and that the flavone skeleton was essential for the suppression of NO and iNOS [69]. Although many types of flavonoids exhibited anti-inflammatory activity, hesperidin and diosmin did not cause significant decreases in NO production in RAW264.7 cells [38].

#### Essential oils

*C.latifolia* Tanaka volatile oil and its main constituent limonene decreased the infiltration of peritoneal exudate leukocytes and the number of polymorphonuclear leukocytes in zymosan-induced peritonitis, and additionally reduced TNF-α levels (but not IL-10 levels) in the peritoneal exudates [70]. Citropten and bergapten from bergamot oil, were found as strong inhibitors of interleukin-8 (IL-8) expression, and could be proposed as potential anti-inflammatory molecules to reduce lung inflammation in patients with cystic fibrosis [71].

#### Coumarins

Auraptene exhibited anti-inflammatory activities by suppressing the production of inflammatory factors that mediated the interaction between adipocytes and macrophages [72]. Another coumarin, imperatorin, also showed anti-inflammatory activity in LPS-stimulated mouse macrophage (RAW264.7) in vitro and a carrageenan-induced mouse paw edema model in vivo [73]. Besides, imperatorin blocked the protein expression of iNOs and COX-2 in LPS-stimulated RAW 264.7cells. 7, 8-dimethoxycoumarin (100 mg/kg) from *C.decumana* peels showed ameliorative effect on gastric inflammation [74].

### Anti-cancer

*Citrus* fruits are high in secondary metabolites, including flavonoids, limonoids, and coumarins, which are associated with a reduced risk of cancer, including gastric cancer, breast cancer, lung tumorigenesis, colonic tumorigenesis, hepatocarcinogenesis, and hematopoietic malignancies, etc. [75–81] Chang and Jia found Ougan (*Citrus reticulata cv. Suavissima*) flavedo extract exhibited potential anti-tumor effects by its inhibitory effect on epithelial-to-mesenchymal transition and interfering with the canonical TGF-β1-SMAD-Snail/Slug axis [82].

#### Flavonoids

Pre- and post-treatment with naringenin effectively suppressed NDEA-initiated heap-tocarcinoma and the associated preneoplastic lesions by modulating xenobiotic-metabolizing enzymes, alleviating lipid peroxidation, and decreasing the levels of liver-marker enzymes [30]. Additionally, naringenin has also been documented in cadmium-induced hepatotoxicity and MNNG-induced gastric carcinogenesis [31, 32]. Supplemented hesperetin to DMH-treated rats suppressed the formation of aberrant crypt foci and significantly reduced the activity of bacterial enzymes in colon cancer [27]. The results clearly revealed that dietary hesperetin possessed antiproliferative ability against chemically-induced colon tumorigenesis [28]. Apigenin was able to cause cell death of BxPC-3 and PANC-1 human pancreatic cancer cells by the inhibition of the GSK-3β/NF-κB signaling cascade leading to the induction of apoptosis [83]. Poncirin showed a significant in vitro inhibitory effect on the growth of the human gastric cancer cells, SGC-7901, in a dose-dependent manner [84]. Tangeretin caused arrest of the cell-cycle progression at the G1 phase and growth inhibition in the incubation of colon adenocarcinoma COLO 205 cells [35]. Quercetin was found to exhibit a suppressive effective in colon carcinogenesis and human cervical cancer cells, but it was found to be ineffective in mammary carcinogenesis [85]. Nobiletin (methoxylated flavonoids) exerted inhibitory effects on the cell adhesion, invasion, and migration abilities of a highly metastatic AGS cells under non-cytotoxic concentrations through Ras/PI3K/AKT signaling pathway [39]. Polymethoxyflavones from *C.tamurana*, *C.tachibana* and *C.kinokuni* show anticancer activity [43] The cytotoxicity of methoxylated flavonoids was higher than that of the hydroxylated analogues [86]. However, it was found that 5-demethylnobiletin exhibited much stronger inhibitory effects on the growth of various cancer cells than nobiletin, suggesting the pivotal role of the hydroxyl group at the 5-position in the enhanced anti-cancer activity [87].

#### Limonoid

Limonoids, including methyl nomilinate, isoobacunoic acid, isolimonexic acid, and limonexic acid, were evaluated for their biological effects on SW480 human colon adenocarcinoma cells [88]. Among them, methyl nomilinate was the most potent inhibitor of cell metabolic activity in MTT and EdU incorporation assays. A study reported that the anti-proliferative properties of limonoids from *C.limon* L. Burm were mediated by caspase-7-dependent pathways in breast cancer cells [89]. Moreover, their cytotoxic effect was more pronounced in estrogen-responsive breast cancer cells. The combinations of limonoids and curcumin were effective in inducing apoptosis in SW480 cells [90]. Furthermore, limonoids and curcumin exhibited synergistic inhibition of proliferation of colon cancer cells, which was supported by the total caspase-3 activity in the cells treated with combinations of limonoids and curcumin.

#### Coumarins

Oltipraz, auraptene, imperatorin, isopimpinellin, and auraptene all significantly increased liver cytosolic GST activities in Nrf2 heterozygous mice, suggesting anti-carcinogenic activities [91]. Besides, 5-geranyloxy-7-methoxycoumarin, limettin, and isopimpinellin inhibited human colon cancer (SW-480) cell proliferation, with 5-geranyloxy-7-methoxycoumar showing the highest inhibition activity (67 %) at 25 µm [92].

#### Carotenoids

β-Cryptoxanthin was reported to inhibit mouse skin tumorigenesis and rat colon carcinogenesis [93].

### Cardiovascular protective effects

Large epidemiological studies frequently link increased consumption of flavonoid-rich foods with reduced cardiovascular morbidity and mortality [94] through the impact on blood lipid, blood glucose and vascular function. Herwandhani Putri found that *Citrus hystrix* kaffir lime’s peel ethanolic extract had potency to be developed as cardioprotector agent in chemotherapy [95].

#### Impact on blood lipid

*Flavonoids* A number of experiments suggested that *Citrus*-derived flavonoids may lower blood cholesterol (CH) and triglyceride (TG). Full methoxylation of the A-ring of *Citrus* flavonoids appeared to be the optimal structure to express potent effects on modulating hepatic lipid metabolism via primarily suppressing apoB-containing lipoprotein secretion using HepG2 cells [96]. Tangeretin and nobiletin, which have the optimal molecular structure, may lower blood CH and TG concentrations, whereas other *Citrus* flavonoids without a fully methoxylated A-ring may have virtually no or only weak lipid-lowering effects in humans such as hesperidin and naringin [36]. In high-fat fed Ldlr^−/−^ mice, the addition of nobiletin resulted in a dramatic reduction in both hepatic and intestinal TG accumulation, attenuation of very low-density lipoprotein(LDL)-TG secretion and normalization of insulin sensitivity [40]. However, a study demonstrated that hydroxylated PMFs, such as 3′,4′-didemethylnobiletin and 5-demethylnobiletin, were more potent than permethoxylated nobiletin in inhibiting PMA-induced scavenger receptor expression and modifying LDL uptake in THP-1 cells [97].

#### Impact on blood glucose

*FlavonoidsCitrus* flavonoids (hesperidin, naringin, neohesperidin, and nobiletin) significantly inhibited amylase-catalyzed starch digestion. Moreover, naringin and neohesperidin mainly inhibited amylose digestion, whereas hesperidin and nobiletin inhibited both amylose and amylopectin digestion. These results demonstrated that *Citrus* flavonoids play important roles in preventing the progression of hyperglycemia, partly by binding to starch, increasing hepatic glycolysis and the glycogen concentration, and lowering hepatic gluconeogenesis [98]. Hesperidin, naringin, and nobiletin also exhibited antidiabetic activities, partly by lowering hepatic gluconeogenesis or improving insulin sensitivity in diabetic animals [99]. A study suggested that naringenin conferred protection against experimental diabetes through its antihyperglycemic and anti-oxidant properties in streptozotocin–nicotinamide-induced experimental diabetic rats [33]. In vivo chronic treatment of diabetic rats with naringenin could prevent the functional changes in vascular reactivity in diabetic rats through a NO-dependent and prostaglandin-independent pathway [34].

#### Impact on vascular function

*Flavonoids* Naringenin and hesperetin might exert anti-atherogenic effects partly through activating peroxisome proliferator-activated receptor and up-regulating adiponectin expression in adipocytes [100]. A study investigated the anti-atherosclerotic action and underlying mechanism of 5-demethylnobiletin in a cell-culture system and determined that 5-demethylnobiletin attenuated monocyte differentiation into macrophage and blunts foam cell formation by down regulating SR expression and activity [97]. This compound also altered the lipid homeostasis in hepatocytes by up-regulating LDL receptor expression via steroid-response element-binding protein-2 activation and down-regulating diacylglycerol acyltransferases 2 expression. In individuals with stage I hypertension, a double-blind crossover trial evaluated the effect on blood pressure of the consumption of a high-flavonoid *Citrus* juice compared to a low-flavonoid *Citrus* juice [101]. Only consumption of the high-flavonoid *Citrus* juice during 5 weeks resulted in a significant reduction in diastolic blood pressure (−3.7 mmHg). However, another controlled crossover trial involving individuals with metabolic syndrome had shown an improvement in flow-mediated dilation after a 3-week supplementation with 500 mg of hesperidin but with no effect on blood pressure [102].

### Neuroprotective effects

In Ming Wu and Hongwu Zhang’s paper, they showed both *C. aurantium* L. aqueous extract and its major constituents (naringin, hesperidin, neohesperidin, and nobiletin) had neuroprotective effect on corticosterone-induced neurotoxicity in PC12 cells. The in vivo and in vitro results suggest that *C. aurantium* L. aqueous extract had an antidepressant effect [103].

#### Flavonoids

The *Citrus* flavanones hesperidin, hesperetin, and neohesperidin have neuroprotective activity against H_2_O_2_-induced cytotoxicity in pheochromocytoma cell line (PC12 cells) by diverse mechanisms, including anti-oxidant activity, regulation of intracellular calciumions, and inhibition of caspase-3 activity [104]. Hwang et al. [105] tested the effect of *Citrus* flavonoids against oxidative stress in PC12 cells, showing neuroprotection by the modulation of Akt/PKB, c-jun N-terminal kinase and P38 activation. Meanwhile, they also found flavonoids acted more as signaling molecules than as anti-oxidants in this study. A pilot clinical study suggested the possibility that 1-year oral administration of decocted nobiletin-rich *C.reticulata* peel could be of benefit for improving the cognition of patients with Alzheimer’s disease, with no adverse side effects [41]. A study showed that 3,5,6,7,8,3′,4′-heptamethoxyflavone had the ability to induce brain-derived neurotrophic factor production in astrocytes and enhance neurogenesis after brain ischemia, which may be mediated by activation of extracellular signal-regulated kinases 1/2 (ERK1/2) and cAMP response element-binding protein [106].

#### Coumarins

Auraptene and 7-isopentenyloxycoumarin exerted protective effects against NMDA-induced excitatory neurotoxicity in mixed cortical cell cultures [107]. Using a transient global ischemia mouse model, a study showed that auraptene effectively inhibited microglia activation, COX-2 expression by astrocytes, and neuronal cell death in the hippocampus following ischemic insults [108]. Auraptene had the ability to induce the activation of ERK1/2 in not only cortical neurons but also the rat PC12 cells and was able to promote neurite outgrowth from PC12 cells.

### Other bioactivities

Apart from widely reported bioactivities mentioned above, other bioactivities of *Citrus* fruits from latest studies have also been reviewed (see Table 1).

**Table 1:** Other bioactivities of *Citrus* fruits reviewed from studies in latest 6 years

Bioactivities	Components	Sources	Subjects	Results	Ref.
Hepatoprotective effects	Citromitin, tangeretin, nobiletin	*C. depressa* juice	d-Galactosamine-treated rats	Suppression on d-galactosamine-induced liver injury	[37]
	Limonin	*Citrus* fruits	d-Galactosamine-treated rats	Attenuation of the markers of hepatic damage and hepatic inflammation Suppression on oxidative stress and expression of TLR-4 but not TLR-2	[109]
Anti-microbial effects	Essential oils	Three *C.* species (orange, lemon, madarin) from Spain	*Enterococcus faecium*, *Staphylococcus aureus,* *Pseudomonas aeruginosa*, and *Salmonella enterica* subsp. *enterica ser.* Enteritidis	Inhibition of spoiling and pathogenic microorganisms	[110]
	Naringenin	*Citrus* fruits	*Salmonella* Typhimurium LT2	Attenuation of Salmonella Typhimurium virulence and cell motility	[111]
	Naringenin, kaempferol, quercetin and apigenin	*Citrus* fruits	*Escherichia coli* O157:H7 and *V. harveyi*	Affection on antagonists of cell–cell signalling Suppression the biofilm formation Alteration the expression of genes encoding type three secretion system in *V. harveyi* ( naringenin)	[112]
Anti-allergic effects	Hesperetin, naringenin	*Citrus* fruits	Rat basophil leukemia RBL-2H3 cells	Inhibition of degranulation by suppression of pathway signals Reduction the symptoms of allergy by inhibiting phosphorylation of Akt	[113]
Anti-melanogenesis effects	Extract	Unripe fruit of *C.* *hassaku*	Cultured murine B16 melanoma cells, the dorsal skin of brownish guinea pigs	Inhibition of melanogenesis without any effects on cell proliferation in cultured murine B16 melanoma cells after glucosamine exposure Prevention effects *in vivo* against UVB-induced pigmentation	[114]
Anti-obesity anti-hyperglycemic effects	Limonoid, nomilin	*Citrus* fruits	Mice fed a high-fat diet	Mediation through the activation of TGR5	[115]
Anti-obesity effects	Extract	Peels of immature *C. sunki*	High-fat diet-induced obese C57BL/6 mice and mature 3T3-L1 adipocytes	Elevation of *β*-oxidation and lipolysis in adipose tissue	[116]
	Polymethoxyflavones, coumarin derivatives	Peels of *Citrus* fruits	Mouse 3T3-L1 preadipocyte cells	Monitoring the prevention of accumulation of lipid droplets	[46]
Inhibitory effects on pulmonary fibrosis	Alkaline	*C.* *reticulata*	Pulmonary fibrosis rats	Inhibition of the proliferation of MRC-5 Prevention effect on bleomycin-induced pulmonary fibrosis in rats	[117]
Anti-diabetic effects	Extract	Yuja (*C. junos* Tanaka) pulp	Mice fed a high fat diet	Reduction the weight gain and the rise in liver fat content, serum triacylglycerol, total cholesterol, and insulin resistance Reduction the secretion of adipocytokines such as leptin and resistin Increase on phosphorylation of AMPK in muscle tissues	[118]
Wound healing effects	Extract	*C. tamurana*	Fibroblasts cells (TIG-119)	Inhibition proliferation of TIG-119 cells at higher concentration (>1.0 mg/mL) Exhibition linear and time-dependent cell proliferation at lower concentrations (0.1, 0.25, 0.5, and 0.75 mg/mL) Acceleration the migration of cells towards the wounded region	[119]
Antianxiety-like effects	Chimpi	Dried *Citrus* peels	ICR male mice	Possession a significant anxiolytic-like effect similar to that of fluoxetine	[120]

The table reviewed latest reported studies concerning other bioactivities of Citrus fruits, including hepatoprotective effects, anti-microbial effects, anti-allergic effects, anti-melanogenesis effects, anti-obesity effects, anti-diabetic effects, etc

## Application of *Citrus* species

*Citrus* species are 131 million tons of fruits produced in 2012 [6]. This large production is also relevant to the high consumption of *Citrus* fruits. Moreover, *Citrus* fruits rank first in international fruit trade in terms of its values of which cover fresh *Citrus* market and processed *Citrus* product market (such as food additives, spices, cosmetic ingredients, juice, jam, and chemotherapeutic drugs).

Given the plentiful bioactivities of *Citrus* fruits, the clinical use of them is of great significance. Investigation among 42,470 Japanese adults showed that *Citrus* consumption was associated with reduced all-cancer incidence, especially for subjects that had simultaneously high green tea consumption [121]. A cross-sectional study (2031 elderly individuals) examined the relationship between the intake of different plant foods and cognitive performance and found *Citrus* fruits had the strongest associations with mean test scores (positively) [122]. Another study found hesperidin (*Citrus* flavonoids) presented a better balance in bone metabolism on bone health [123]. And immature peels of citrus fruit are used to treat indigestion and have demonstrated potential as a chemotherapeutic agent [124, 125]. Many other studies have also shown that the consumption of *Citrus* fruits is associated with inhibition of various cancers, including colorectal, esophageal, and stomach cancer, as well as anti-stroke activity, improved blood lipid profiles and improved survival of the elderly [16]. And more and further studies are still required for *Citrus* species as chemotherapeutic drugs.

The consumption of Citrus fruits or juice is inversely associated with several diseases because of its abundant secondary metabolites. Almost 33 % of the Citrus fruits are industrially processed for juice production, however, where about half of processed Citrus including peels, segment membrane and seeds end up as wastes [126]. These solid residues are referred to as Citrus wastes with estimated worldwide production of 15 million tons per year [127]. What’s more, as reviewed in additional files, these Citrus wastes are still rich in various biologically secondary metabolites associated with human health. Citrus peel contains a high content of polymethoxylated flavones and flavanones, including primarily hesperidin, nobiletin, neohesperidin, naringin and tangeretin. A study suggested that hesperetin could be exploited as a potential functional ingredient and offered opportunities to develop new formulations of functional foods [27]. Peels are also major source of essential oil as well as carotenoids, with approximately 70 % of the total fruit carotenoids, and their contents may be from two to six times higher than those of the endocarp [128]. Besides, seeds are the major sources of limonoids. Mayumi Minamisawa et al. have succeeded in extracting a large amount of limonoids from yuzu (*Citrus junos*) seeds which contain higher amounts of fat-soluble limonoid aglycone (330.6 mg/g of dry seed), water-soluble limonoid glycoside (452.0 mg/g of dry seed), and oil (40 mg/g of green seed) [129]. *Citrus* species are noticeably beneficial fruits for consumption daily both for their nutrients contents and multiple active metabolites with related bioactivities, which manifests it is worthwhile to develop more useful recycling approaches of Citrus wastes. The applications given by Citrus wastes may help the industrial processors to find new ways of increasing the profit by recycling bioactive compounds and also reducing the considerable problem of wastes.

## Conclusion and prospective

The multiple secondary metabolites in *Citrus*, including flavonoids, alkaloids, coumarins, limonoids, carotenoids, phenolic acids and volatile compounds, provide a rational basis for various biological activities. Among them, flavonoids (especially flavanones, flavanonols and methoxylated flavones) exhibit more bioactivities compared to other secondary metabolites. However, all these active metabolites work synergistically to exhibit anti-oxidative, anti-inflammatory, anti-cancer, anti-microbial and anti-allergy effects, as well as presenting cardiovascular protection, neuroprotective effect, hepatoprotective effect, etc. Consequently, these multiple active metabolites with various bioactivities indicate that *Citrus* species are beneficial fruits when eaten daily, both for their nutrients contents and as chemotherapeutic or complementary medicine to promote health. Furthermore, different species, fruit parts, stages of maturity, environmental conditions during growth, storage conditions and postharvest treatments can influence the level of active metabolites and related activities. And further investigations are required in order to make optimal use of these fruits.

## Additional files

10.1186/s13065-015-0145-9 Flavonoids isolated from *Citrus* species. The table summarized flavones (including polymethoxylated flavones), flavonols, flavanones and flavanonols from *Citrus* species including *C. aurantifolia*, *C. aurantium*, *C. canaliculata*, *C. clementina*, *C. erythrosa*, *C. grandis*, *C. hassaku*, *C. hystrix*, *C. junos*, *C. kinokuni*, *C. leiocarpa*, *C*. *limon*, *C. limonimedica*, *C. medica*, *C. microcarpa*, *C*. *paradisi*, *C. reticulate*, *C*. *sinensis*, *C*. *suhuiensis*, *C. tachibana*, *C. tamurana* and *C*. *unshiu.*

10.1186/s13065-015-0145-9 Alkaloids, coumarins, limonoids, carotenoids and phenolic acids isolated from *Citrus* species. The table summarized alkaloids, coumarins, limonoids, carotenoids and phenolic acids from *Citrus* species including *C. aurantifolia*, *C. aurantium*, *C. bergamia*, C. canaliculata, *C. clementina*, *C. grandis*, *C. hassaku*, *C. junos*, *C. kinokuni*, *C. leiocarpa*, *C*. *limon*, *C. limonimedica*, *C. maxima*, *C. microcarpa*, *C. myrtifolia*, *C*. *paradisi*,, *C. reticulate*,*C*. *sinensis*, *C. tachibana* and *C*. *unshiu.*

10.1186/s13065-015-0145-9 Volatile compounds isolated from *Citrus* species. The table summarized citrus-derived volatile compounds from common *Citrus* Species including *C. Aurantium, C. Aurantifolia, C. Medica, C. Limon, C. Bergamia, Citrus reticulata, C. Kinokuni, C. Unshiu, C. Clementina, C. Sinensis, C. Clementine* × *C. Tangerine, C. grandis* × *C. Grandis, C. Paradisi, C. Nobilis* and *C. depressa.*

## Abbreviations

PMF: polymethoxylated flavones
ROS: reactive oxygen species
LPS: lipopolysaccharide
TNF-α: tumor necrosis factor-alpha
iNOS: inducible nitric oxide synthase
COX-2: cyclooxygenase-2
NO: nitric oxide
CH: cholesterol
TG: triglyceride
LDL: low-density lipoprotein
