# Supplementary material for: Citrus fruits as a treasure trove of active natural metabolites that potentially provide benefits for human health
Source: Chem Cent J. 2015 Dec 24;9:68. doi: 10.1186/s13065-015-0145-9 (PMC4690266; doi:10.1186/s13065-015-0145-9)
Supplement: Supplementary file 1 — 10.1186/s13065-015-0145-9 Flavonoids isolated from Citrus species. The table summarized flavones (including polymethoxylated flavones), flavonols, flavanones and flavanonols from Citrus species including C. aurantifolia, C. aurantium, C. canaliculata, C. clementina, C. erythrosa, C. grandis, C. hassaku, C. hystrix, C. junos, C. kinokuni, C. leiocarpa, C. limon, C. limonimedica, C. medica, C. microcarpa, C. paradisi, C. reticulate, C. sinensis, C. suhuiensis, C. tachibana, C. tamurana and C. unshiu. [file 13065_2015_145_MOESM1_ESM.pdf]

Additional file 1 Flavonoids isolated from *Citrus* species.

compounds	Structure	source	ref.
flavanone and flavanonol	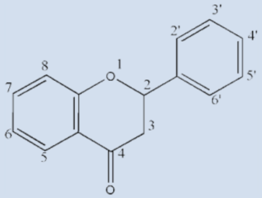		
naringenin	5-OH, 4'-OH, 7-OH	CJ[a]; CJ[b]; CG[a]; CG[b]	[1]
hesperetin	5-OH, 3'-OH, 4'-OH, 7-OCH_3	CJ[a]; CJ[b]; CAF[d]	[1, 2]
narirutin	5-OH, 4'-OH, 7-O-rut.	CSI[a]; CSI[b]; CU[a]; CU[b]; CLI[b]; CP[b]; CSU[b]; CR[a]; CR[b]; CCL[a]; CAT[d]	[3, 4, 5, 6, 7]
naringin	5-OH, 4'-OH, 7-O-neo.	CAT[a]; CAT[d]; CJ[a]; CJ[b]; CU[a]; CU[b]; CG[a]; CG[b]; CG[e]; CP[b]; CR[d]; CAT[a]	[3, 5, 8, 9, 10]
naringenin-7-O-rutinoside-4'-O-glucoside	5-OH, 7-O-rut., 4'-O-glu.	CSI[b]; CR[b]; CU[b]; CP[b]; CAT[d]	[3, 5]
naringenin-7-O-neohesperidoside-4'-O-glucoside	5-OH, 7-O-neo., 4'-O-glu.	CP[b]	[3, 5]
naringin dehydrated hexoside	5-OH, 4'-O-dih., 7-O-neo.	CAT[a]	[11]
hesperidin	5-OH, 3'-OH, 4'-OCH_3, 7-O –rut.	CR[a]; CR[b]; CR[d]; CCL[a]; CU[a]; CU[b]; CSI[a]; CSI[b]; CLI[b]; CP[b]; CSU[a]; CSU[b]; CMI[a]; CMI[b]; CME[a]; CME[b]; CHY[a]; CHY[b]; CLE[a]; CAT[a]; CAT[d]; CE[a]; CJ[a]; CJ[b]; CG[a]; CG[b]; CG[e] ; CAF[d]	[1, 2, 3, 4, 6, 7, 8, 10]
neohesperidin	5-OH, 3'-OH, 4'-OCH_3, 7-O-neo.	CP[b]; CMI[a]; CMI[b]; CME[a]; CME[b]; CHY[a]; CHY[b]; CJ[b]; CU[a]; CU[b]; CG[a]; CG[b]; CG[e]; CAT[d]; CAT[a]	[1, 3, 5, 9, 10]
hesperidin dehydrated hexoside	5-OH, 4'-OCH_3, 3'-dih., 7-O-rut.	CAT[a]	[8]
eriocitrin	5-OH, 3'-OH, 4'-OH, 7-O-rut.	CSI[b]; CR[b]; CU[b]; CLI[b]; CME[a]; CME[b]; CHY[b]; CAT[d]	[3, 5, 10]
neoeriocitrin	5-OH, 3'-OH, 4'-OH, 7-neo.	CHY[b]; CAT[d]; CAT[a]	[5, 9, 10]

eriodictyol-7-O-rutinoside-4'-O-glucoside	5-OH, 3'-OH, 4'-O-glu., 7-O-rut.	CLI[b]	[3]
didymin	5-OH, 4'-OCH_3, 7-O-rut.	CSI[a]; CSI[b]; CU[a]; CU[b]; CLI[b]; CP[b] CSU[a]; CSU[b]; CR[a]; CR[b]; CCL[a]; CAF[d]	[3, 6, 10, 12]
poncirin	5-OH, 4'-OCH_3, 7-O-neo.	CP[b]; CAT[a]; CR[a]; CR[b]	[3, 11, 13]
flavone and flavonol	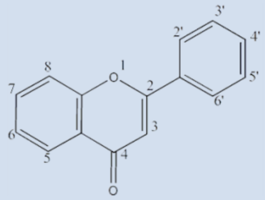		
luteolin	5-OH, 4'-OH, 5'-OH, 7-OH	CJ[a]; CJ[b]; CU[a]; CU[b]; CG[a]; CG[b]	[1]
apigenin	5-OH, 4'-OH, 7-OH	CAT[d]	[10]
quercetin	3-OH, 5-OH, 7-OH, 4'-OH, 5'-OH	CR[d]; CAT[d]	[10]
luteolin-7-O -rutinoside	5-OH, 4'-OH, 5'-OH, 7-O-rut.	CR[b]; CU[b]; CLI[b]	[3]
luteolin-7-O-neohesperidoside	5-OH, 4'-OH, 5'-OH, 7-O- neo.	CAT[a]	[9]
luteolin-6,8-di-C -glucoside	5-OH, 4'-OH, 5'-OH, 7-OH, 6-C-glu., 8-C-glu.	CSI[b]; CLI[b]; CAT[d]	[3, 5]
luteolin-7-O-neohesperidpode-4'-O-glucoside	5-OH, 4'-O-glu.e, 5'-OH, 7-O-neo.	CP[b]	[3]
apigenin-7-O -rutinoside	5-OH, 4'-OH, 7-O-rut.	CR[b]; CU[b]; CLI[b]	[3]
apigenin-7-O-neohesperidoside	5-OH, 4'-OH, 7-O-neo.	CP[b]; CAT[d]	[3, 5]
apigenin-6,8-di-C-glucoside	5-OH, 4'-OH, 7-OH, 6-C-glu., 8-C-glu.	CSI[b]; CR[b]; CU[b]; CLI[b]; CP[b]; CSU[a]; CSU[b]; CMI[b]; CAT[d]; CAT[a]	[3, 4, 5, 9]
apigenin-8-C-glucosyl-2''-O-rhamnoside	5-OH, 4'-OH, 7-OH, 8-C-glul., -2''-O-rha.	CMI[a]; CMI[b]	[4]
apigenin-7-O-rutinoside-4'-O-glucoside	5-OH; 4'- O-glu., 7-O –rut.	CLI[b]	[3]
apigenin-7-O-neohesperidoside-4'-glucoside	5-OH, 4'-O-glu., 7-O- neo.	CAT[a]	[9]
chrysoeriol-7-O-rutinoside	5-OH, 4'-OH, 5'-OCH_3, 7-O-rut.	CR[b]; CU[b]; CLI[b]	[3]
chrysoeriol-6,8-di-C-glucoside	5-OH, 4'-OH, 5'-OCH_3, 7-OH, 6-C-glu., 8-C-glu.	CLI[b]; CSU[a]	[3, 4]

diosmin	5-OH, 3'-OH, 4'-OCH_3, 7-O-rut.	CR[b]; CU[a]; CU[b]; CLI[b]; CME[a]; CME[b]; CHY[a]; CCL[a]; CSI[a]	[3, 4, 6]
diosmetin-8-C -glucoside	5-OH, 3'-OH, 4'-OCH_3, 7-OH, 8-C-glu.	CR[b]; CU[b]; CLI[b]	[3]
diosmetin-6- C-glucoside	5-OH, 3'-OH, 4'-OCH_3, 7-OH, 6-C-glu.	CR[b]; Cu[b]; CLI[b]; CME[b]	[3, 4]
diosmetin-6,8-di-C-glucoside	5-OH, 3'-OH, 4'-OCH_3, 7-OH, 6-C-glu., 8-C-glu.	CR[b]; CU[b]; CLI[b]; CSU[a]; CME[a]; CME[b]; CAT[a]	[3, 4, 9]
rutin	3-O-rut., 5-OH, 7-OH, 4'-OH, 5'-OH	CSI[b]; CR[b]; CR[d]; CU[b]; CLI[b]; CAT[d]; CAF[d]	[3, 10, 12]
quercetin-7-O -rutinoside	3-OH, 5-OH, 7-O-rut., 4'-OH, 5'-OH	CSI[b]; CR[b]; CU[b]; CLI[b]; CP[b]	[3]
isorhamnetin-3-O-rutinoside	5-OH, 3-O-rut., 7-OH, 4'-OH, 5'-OCH_3	CSI[b]; CR[b]; CU[b]; CLI[b]	[3]
isorhamnetin-7-O-rutinoside	5-OH, 3-OH, 7-O-rut, 4'-OH, 5'-OCH_3	CSI[b]; CLI[b]	[3]
tamarixetin-7-O -rutinoside	3-OH, 5-OH, 4'-OCH_3, 5-OH, 7-O -rut.	CR[b]; CU[b]	[3]
kaempferol-3-O -rutinoside	5-OH, 3-O-rut., 7-OH, 4'-OH	CSI[b]; CR[b]; CU[b]	[3]
kaempherol	5-OH, 3-O-glu., 7-O-glu. 4'-OH	CAT[d]	[10]
Polymethoxylatedflavones	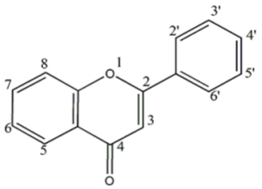		
sinensetin	5-OCH_3, 6-OCH_3, 7-OCH_3, 4'-OCH_3, 5'-OCH_3	CAT[a]; CE[a]; CCL[a]; CU[a]; CSI[a]; CR[a]; CR[b]; CLM[a]; CSI[a]; CTR[a]; CU[a]; CK[a]; CTB[a]	[6, 7, 11, 14]
isosinensetin	3'-OCH_3, 4'-OCH_3, 5-OCH_3, 7-OCH_3, 8-OCH_3	CLE[a]; CE[a]	[11]
nobiletin	3'-OCH_3, 4'-OCH_3, 3-OCH_3, 5-OCH_3, 6-OCH_3, 7-OCH_3	CLE[a]; CAT[a]; CE[a]; CCL[a]; CU[a]; CSI[a]; CR[a]; CR[d]; CP[a]; CP[c]; CHA[a]; CSI[a]; CTR[a]; CK[a]; CTB[a]	[6, 7, 9, 11, 14]
tangeretin	5-OH, 6-OCH_3, 7-OCH_3, 8-OCH_3, 4'-OCH_3	CLE[a]; CAT[a;] CE[a]; CCL[a;] CU[a;] CSI[a]; CR[a]; CR[b]; CHA[a]; CSI[a]; CTR[a]; CU[a]; CK[a]; CTB[a];	[6, 7, 9, 11, 14]
3,5,6,7,8,3',4'-heptamethoxyflavone	3-OCH_3, 5-OCH_3, 6-OCH_3, 7-OCH_3,	CHA[a]; CSI[a]; CTR[a]; CU[a]; CK[a]; CTB[a]; CLE[a]; CP[a]; CP[c]	[14]

	8-OCH_3, 3'-OCH_3, 4'-OCH_3		
5-hydroxy-6,7,8,3',4'-pentamethoxyflavone	5-OH, 6-OCH3, 7-OCH3, 8-OCH3, 3'-OCH3, 4'-OCH3	CLM[a]; CHA[a]; CCA[a]; CSI[a]; CU[a]; CK[a]; CTB[a]; CLE[a]	[14]
5,7,8,3',4'-pentamethoxyflavone	5-OCH3, 7-OCH3, 8-OCH3, 3'-OCH3, 4'-OCH3	CSI[a]; CTR[a]; CU[a]; CK[a]; CTB[a]; CLE[a]	[14]

dih: dihydratedhexoside; glu: glucoside; glul: glucosyl; rha: rhamnoside; rut: rutinoside; neo: neohesperidoside; [a]: peel; [b]: pulp; [c]: pressed oil; [d]: juice; [e]: whole fruit.;CAF: *C. aurantifolia*; CAT: *C. aurantium*; CCA: *C. canaliculata*; CCL: *C. clementina*; CE: *C. erythrosa*; CG: *C. grandis*; CHA: *C. hassaku*; CHY: *C. hystrix*; CJ: *C. junos*; CK: *C. kinokuni*; CLE: *C. leiocarpa*; CLI: *C. limon*; CLM: *C. limonimedica*; CME: *C. medica*; CMI: *C. microcarpa*; CP: *C. paradisi*; CR: *C. reticulate*; CSI: *C. sinensis*; CSU: *C. suhuiensis*; CTB: *C. tachibana*; CTR: *C. tamurana*; CU: *C. unshiu*.

1. Yoo KM, Hwang IK, Park JH, Moon B: **Major Phytochemical Composition of 3 Native Korean Citrus Varieties and Bioactive Activity on V79 - 4 Cells Induced by Oxidative Stress.** *J Food Sci* 2009, **74**: C462-C468.

2. Patil JR, Chidambara Murthy K, Jayaprakasha G, Chetti MB, Patil BS: **Bioactive compounds from Mexican lime (Citrus aurantifolia) juice induce apoptosis in human pancreatic cells.** *J Agric Food Chem* 2009, **57**: 10933-10942.

3. Abad-Garcia B, Berrueta LA, Garmon-Lobato S, Urkaregi A, Gallo B, Vicente F: **Chemometric characterization of fruit juices from Spanish cultivars according to their phenolic compound contents: I. Citrus fruits.** *J Agric Food Chem* 2012, **60**: 3635-3644.

4. Roowi S, Crozier A: **Flavonoids in tropical citrus species.** *J Agric Food Chem* 2011, **59**: 12217-12225.

5. Barreca D, Bellocco E, Caristi C, Leuzzi U, Gattuso G: **Distribution of C- and O-glycosyl flavonoids,(3-hydroxy-3-methylglutaryl) glycosyl flavanones and**

furocoumarins in Citrus aurantium L juice. *Food Chem* 2011, **124**: 576-582.

6. Bermejo A, Llosa MJ, Cano A: **Analysis of bioactive compounds in seven citrus cultivars.** *Food Sci Technol Int* 2011, **17**: 55-62.

7. Stuetz W, Prapamontol T, Hongsibsong S, Biesalski HK: **Polymethoxylated flavones, flavanone glycosides, carotenoids, and antioxidants in different cultivation types of tangerines (Citrus reticulata Blanco cv. Sainampueng) from Northern Thailand.** *J Agric Food Chem* 2010, **58**: 6069-6074.

8. Kim KN, Heo SJ, Yoon WJ, Kang SM, Ahn G, Yi TH, Jeon YJ: **Fucoxanthin inhibits the inflammatory response by suppressing the activation of NF-κB and MAPKs in lipopolysaccharide-induced RAW 264.7 macrophages.** *Eur J Pharmacol* 2010, **649**: 369-375.

9. Mencherini T, Campone L, Piccinelli AL, Mesa MG, Sanchez DM, Aquino RP, Rastrelli L: **HPLC-PDA-MS and NMR characterization of a hydroalcoholic extract of Citrus aurantium L. var. amara peel with antiedematogenic activity.** *J Agric Food Chem* 2013, **61**: 1686-1693.

10. Moulehi I, Bourgou S, Ourghemmi I, Tounsi MS: **Variety and ripening impact on phenolic composition and antioxidant activity of mandarin (Citrus reticulate Blanco) and bitter orange (Citrus aurantium L.) seeds extracts.** *Ind Crop Prod* 2012, **39:** 74-80.

11. Kim HG, Kim GS, Park S, Lee JH, Seo ON, Lee SJ, Kim JH, Shim JH, Abd El-Aty A, Jin JS: **Flavonoid profiling in three citrus varieties native to the Republic of Korea using liquid chromatography coupled with tandem mass spectrometry: contribution to overall antioxidant activity.** *Biomed Chromatogr* 2012, **26**: 464-470.

12. Patil JR, Chidambara Murthy KN, Jayaprakasha GK, Chetti MB, Patil BS: **Bioactive compounds from Mexican lime (Citrus aurantifolia) juice induce apoptosis in human pancreatic cells.** *J Agric Food Chem* 2009, **57**: 10933-10942.

13. Zhu X, Luo F, Zheng Y, Zhang J, Huang J, Sun C, Li X, Chen K: **Characterization, purification of poncirin from edible citrus ougan (Citrus reticulate cv. suavissima) and its growth inhibitory effect on human gastric cancer cells SGC-7901.** *Int J Mol Sci* 2013, **14**:8684-8697.

14. Hirata T, Fujii M, Akita K, Yanaka N, Ogawa K, Kuroyanagi M, Hongo D: **Identification and physiological evaluation of the components from Citrus fruits as potential drugs for anti-corpulence and anticancer.** *Bioorgan Med Chem* 2009, **17**: 25-28.
