# Supplementary material for: Citrus fruits as a treasure trove of active natural metabolites that potentially provide benefits for human health
Source: Chem Cent J. 2015 Dec 24;9:68. doi: 10.1186/s13065-015-0145-9 (PMC4690266; doi:10.1186/s13065-015-0145-9)
Supplement: Supplementary file 2 — 10.1186/s13065-015-0145-9 Alkaloids, coumarins, limonoids, carotenoids and phenolic acids isolated from Citrus species. The table summarized alkaloids, coumarins, limonoids, carotenoids and phenolic acids from Citrus species including C. aurantifolia, C. aurantium, C. bergamia, C. canaliculata, C. clementina, C. grandis, C. hassaku, C. junos, C. kinokuni, C. leiocarpa, C. limon, C. limonimedica, C. maxima, C. microcarpa, C. myrtifolia, C. paradisi,, C. reticulate,C. sinensis, C. tachibana and C. unshiu. [file 13065_2015_145_MOESM2_ESM.pdf]

Additional file 2 Alkaloids, coumarins, limonoids, carotenoids and phenolic acids isolated from *Citrus* species.

Compounds	source	ref.
alkaloids		
(±)octopamine	CAT[e]; CAT[a]; CR[a]	[1, 2, 3, 4]
(±)synephrine	CAT[e]; CAT[a]; CAT[d]; CR[a]; CU[a]; CSI[e]; CSI[d]	[1, 2, 3, 4, 5]
tyramine	CAT[e]; CR[a]	[2, 3]
N-methyltyramine	CAT[e]; CR[a]	[2, 3]
hordenine	CAT[e]	[2]
γ-aminobutyric acid	CMY[d]; CP[d]; CAT[d]	[6]
2-hydroxyethyltrimethy-lammonium	CMY[d]; CP[d]; CAT[d]	[6]
N-methylnicotinic acid	CMY[d]; CP[d]; CAT[d]	[6]
L-proline	CMY[d]; CP[d]; CAT[d]	[6]
N-methyl-L-proline	CMY[d]; CP[d]; CAT[d]	[6]
N,N-dimethyl-L-proline	CMY[d]; CP[d]; CAT[d]	[6]
4-hydroxy-L-proline	CMY[d]; CP[d]; CAT[d]	[6]
4-hydroxy-L-prolinebetaine	CMY[d]; CP[d]; CAT[d]	[6]
tryptophan	CB[a]; CB[b]; CB[f]	[7]
tryptamine	CB[f]	[7]
N-methyltryptamine	CB[b]	[7]
N,N-dimethyltryptamine	CB[a]; CB[b]	[7]
N,N,N-triethyltryptamine	CB[a]; CB[b]; CB[f]	[7]
5-hydroxytryptamine	CB[f]	[8]
5-hydroxy-N-methyltryptamine	CB[b]; CB[f]	[8]
5-hydroxy-N,N-dimethyltryptamine	CB[a]; CB[b]; CB[f]	[8]
5-hydroxy-N,N,N-trimethyltryptamine	CB[a]; CB[b]; CB[f]	[8]
coumarins		
pranferin	CP[a]; CP[c]	[9]
meranzin	CP[a]; CP[c]; CAT[c]	[9, 10, 11]
isomeranzin	CP[c]; CG[e]; CAT[c]; CAT[a]	[10, 11, 12]
meranzin hydrate	CAT[e]; CAT[a]; CP[c]	[11, 12, 13]
auraptene	CP[a]; CP[c] ;CP[e]; CAT[e]; CMA[a]; CAF[e]; CB[e]; CSI[e]; CR[e]; CLI[e]; CLI[a]; CLM[a]; CHA[a]; CCA[a]	[9, 13, 14, 15, 16]
ecxyaurapten	CP[a]; CMA[a]	[15]
osthol	CP[a]; CP[c]; CMA[a]; CG[e]	[9, 11, 15, 17]
marmin	CP[a]; CP[c]; CAT[e]	[9, 13]
limettin	CSI[a]; CLI[a]; CLI[c]; CP[a]; CP[d]; CB[a]; CB[c]; CMA[a]; CCL[a]; CAF[a]; CAF[c]; CAF[d]; CAF[e]	[10, 15, 18]
5-geranyloxy-7-methoxycoumarin	CLI[a]; CLI[c]; CP[a]; CAF[c]; CAF[e]; CB[c]	[10, 11, 15, 18]
5-isoantenyloxy-7-methoxy-coumarin	CLI[c]	[11]
ecxybergamottin hydrate	CP[c]	[11]
8-geranyloxypsoralen	CLI[a]; CLI[c]; CAF[c]	[10, 15]
umbelliferone	CP[e]; CAF[e]; CB[e]; CAT[e]; CSI[e]; CR[e]; CLI[e]	[16]
herniarin	CAF[c]	[10]

oxyaucedanin	CCL[a]; CLI[a]; CLI[c]; CAF[c]	[10, 11, 15]
oxyaucedanin hydrate	CLI[a]; CLI[c]; CAF[c]	[10, 15]
psoralen	CB[a]	[15]
isopimpinellin	CCL[a]; CLI[c]; CAF[c]; CAF[d]; CAF[a]; CAF[e]	[10, 11, 15, 18, 19]
byakangelicin	CCL[a]	[10]
byakangelicol	CCL[a]; CLI[c]; CAF[c]	[10, 15]
imaratorin	CLI[c]; CAF[c]	[10]
iscmaratorin	CLI[c]; CAF[c]	[10]
cnidilin	CLI[c]; CAF[c]	[10]
cnidicin	CLI[c]; CAF[c]	[10]
bergapten	CP[a]; CP[c]; CCL[a]; CB[a]; CB[c]; CMA[a]; CAF[c]; CAT[c]; CG[e]	[9, 10, 15, 20]
5-hydroxyfurocoumarin	CG[e]	[20]
bergamottin	CLI[a]; CLI[c]; CP[a]; CP[d]; CB[a]; CMA[a]; CAF[c]; CAF[d]; CAF[a]	[10, 11, 15, 19]
ecxybergamottin	CP[a]; CP[c]; CMA[a]; CAT[c]; CAT[d]	[10, 15, 21]
6',7'-dihydroxybergamottin	CP[a]; CP[c]; CMA[a]; CCA[a]; CK[a]; CTB[a]; CLE[a]	[9, 14, 15]
limonoids		
limonin	CG[e]; CR[f]; CR[e]; CMA[e]; CAT[e]; CAT[f]; CAF[d]; CJ[f]	[3, 22, 23, 24, 25, 26, 27]
nomilin	CG[e]; CR[f]; CR[e]; CMA[e]; CAT[e]; CJ[f]; CAT[f]	[3, 23, 24, 25, 26, 27]
obacunone	CR[f]; CJ[f]; CAT[f]	[25, 26, 27]
obacunone acetate	CAT[f]	[27]
deacetyl-nomilin	CAT[e]; CAT[f]	[24, 27]
citriolide-A	CR[f]	[26]
deoxylimonin	CAT[f]	[27]
methyldeacetylnomilinate	CAT[f]	[27]
ichangin	CAT[e]; CAT[f]	[24, 27]
ichangensin	CAT[f]	[27]
limonexic acid	CAF[d]; CLI[f]	[22,25]
isolimonexic acid	CAT[e]; CAF[d]; CLI[f]	[22, 24, 25]
calamin	CAT[f]	[27]
limonin glucoside	CMA[e]; CR[e]; CAT[e]; CAF[d]; CLI[f]; CSI[e]	[22, 23, 24, 25, 28]
nomilin glucoside	CSI[e]	[28]
obacunone glucoside	CMA[e]; CR[e]; CR[f]; CAT[e]; CAT[f]; CLI[f]; CSI[e]	[23, 24, 25, 27, 28]
deacetyl nomilin glucoside	CMA[e]; CR[e]; CAT[e]; CLI[f]; CSI[e]	[23, 24, 25, 28]
nomilinic acid glucoside	CMA[e]; CR[e]; CAT[e]; CLI[f]; CSI[e]	[23, 24, 25, 28]
deacetyl nomilinic acid	CSI[e]	[28]
isoobacuncc acid glucoside	CAT[e]	[24]
obacuncc acid glucoside	CAT[f]	[27]
calamin glucoside	CAT[f]	[27]
carotenoids		
violaxanthin	CCL[a]; CCL[b]; CU[a]; CSI[a]	[29, 30, 31, 32]
β-crytoxanthin	CCL[a]; CCL[b]; CU[a]; CSI[a]	[29, 30, 31, 32]

carotene	CCL[a]; CCL[b]; CU[a]; CSI[a]	[29, 31, 32]
lutein	CCL[b]; CSI[a]	[31, 32]
zeaxanthin	CCL[b]; CSI[a]	[31, 32]
antheraxanthin	CCL[b]; CSI[a]	[30, 31]
cryptoxanthin	CCL[b]; CSI[a]	[31, 32]
phytoene	CCL[b]; CSI[a]	[30, 31, 32]
phytofluene	CCL[b]; CSI[a]	[30, 31, 32]
β-citraurin/accarotencd	CSI[a]	[30, 32]
ζ-Carotene	CCL[b]	[31]
neoxanthin	CSI[a]	[32]
Phenolic acid		
sinapic acid	CU[a]; CMI[a]; CP[a]; CP[b]; CSI[e]	[33, 34, 35, 36]
P-coumaric acid	CAT[d]; CR[a]; CSI[a]; CU[a]; CMI[a]; CP[a]; CP[b]; CSI[e]; CAT[a]; CAT[d]	[33, 34, 35, 36, 37, 38, 39, 40]
ferulic acid	CR[d]; CR[a]; CU[a]; CMI[a]; CP[a]; CP[b]; CSI[e]; CAT[a]; CAT[d]	[33, 34, 35, 36, 37, 39, 40]
caffeic acid	CR[d]; CSI[a]; CU[a]; CMI[a]; CP[a]; CP[b]	[33, 34, 36, 37, 38]
trans-2-hydroxicinnamicacid	CR[d]; CAT[d]; CAT[a]	[37, 40]
trans-cinnamic acid	CAT[d]; CAT[a]	[41]
rosmarinic acid	CR[d]; CAT[d]; CAT[a]	[37, 41]
protocatechuic acid	CU[a]; CP[a]; CP[b]	[34, 36]
P-hydroxybenzcc	CR[a]; CU[a]; CP[a]; CP[b]	[34, 36, 39]
vanillic acid	CR[d]; CR[a]; CAT[a]; CAT[d]; CU[a]; CP[a]; CP[b]	[37, 34, 36, 39, 41]
gallic acid	CR[d]; CR[a]; CAT[a]; CAT[d]	[37, 39, 41]
chlorogenic acid	CR[d]; CP[a]; CP[b]; CAT[a]; CAT[d]	[37, 36]
ferulic-O-hexoside	CSI[b]; CR[b]; CU[b]; CLI[b]; CP[b]	[42]
sinapic-O-hexoside	CSI[b]; CR[b]; CU[b]; CLI[b]	[42]
syringic acid	CR[d]; CAT[a]; CAT[d]	[37, 41]

[a]: peel; [b]: pulp; [c]: pressed oil; [d]: juice; [e]: whole fruit; [f]: seed; CAF: *C. aurantifolia*; CAT: *C. aurantium*; CB: *C. bergamia*; CCA: C. canaliculata; CCL: *C. clementina*; CG: *C. grandis*; CHA: *C. hassaku*; CJ: *C. junos*; CK: *C. kinokuni*; CLE: *C. leiocarpa*; CLI: *C. limon*; CLM: *C. limonimedica*; CMA: *C. maxima*; CMI: *C. microcarpa*; CMY: *C. myrtifolia*; CP: *C. paradisi* peel; CR : *C. reticulate*; CSI :*C. sinensis*; CTB: *C. tachibana*; CU: *C. unshiu*.

1. Nelson BC, Putzbach K, Sharpless KE, Sander LC: **Mass spectrometric determination of the predominant adrenergic protoalkaloids in bitter orange (Citrus aurantium).** *J Agr Food Chem* 2007, **55**: 9769-9775.

2. Percy DW, Adcock JL, Conlan XA, Barnett NW, Gange ME, Noonan LK, Henderson LC, Francis PS: **Determination of Citrus aurantium protoalkaloids using HPLC with acidic potassium permanganate chemiluminescence detection.** *Talanta* 2010, **80**: 2191-2195.

3. Pellati F, Benvenuti S: **Fast high-performance liquid chromatography analysis of phenethylamine alkaloids in Citrus natural products on a pentafluorophenylpropyl stationary phase.** *J Chromatogr A* 2007, **1165**: 58-66.

4. Roman MC, Betz JM, Hildreth J: **Determination of synephrine in bitter orange raw materials, extracts, and dietary supplements by liquid chromatography with ultraviolet detection: single-laboratory validation.** *J AOAC Int* 2007, **90**: 68-81.

5. Mattoli L, Cangi F, Maidecchi A, Ghiara C, Tubaro M, Traldi P: **A rapid liquid chromatography electrospray ionization mass spectrometry(n) method for evaluation of synephrine in Citrus aurantium L. samples.** *J Agric Food Chem* 2005, **53**: 9860-9866.

6. Servillo L, Giovane A, Balestrieri ML, Bata-Csere A, Cautela D, Castaldo D: **Betaines in fruits of Citrus genus plants.** *J Agric Food Chem* 2011, **59**: 9410-9416.

7. Servillo L, Giovane A, Balestrieri ML, Cautela D, Castaldo D: **N-methylated tryptamine derivatives in citrus genus plants: identification of N,N,N-trimethyltryptamine in bergamot.** *J Agric Food Chem* 2012, **60**: 9512-9518.

8. Servillo L, Giovane A, Balestrieri ML, Casale R, Cautela D, Castaldo D: **Citrus genus plants contain N-methylated tryptamine derivatives and their 5-hydroxylated forms.** *J Agric Food Chem* 2013, **61**: 5156-5162.

9. Chebrolu KK, Jayaprakasha GK, Jifon J, Patil BS: **Purification of coumarins, including meranzin and pranferin, from grapefruit by solvent partitioning and a hyphenated chromatography.** *Sep Purif Technol* 2013, **116:** 137–144.

10. Dugo P, Piperno A, Romeo R, Cambria M, Russo M, Carnovale C, Mondello L: **Determination of oxygen heterocyclic components in citrus products by HPLC with UV detection.** *J Agric*

Food Chem 2009, **57**: 6543-6551.

11. Marina Russo GT, Caterina Camovale, Ivana Bonaccorsi, Luigi Mondello, Paola Dugo: **A new HPLC method developed for the analysis of oxygen heterocyclic compounds in Citrus essential oils.** *J Essent Oil Res* 2012, **24**: 119-129.

12. Mencherini T, Campone L, Piccinelli AL, Mesa MG, Sanchez DM, Aquino RP, Rastrelli L: **HPLC-PDA-MS and NMR characterization of a hydroalcoholic extract of Citrus aurantium L. var. amara peel with antiedematogenic activity.** *J Agric Food Chem* 2013, **61**: 1686-1693.

13. Chen HF, Zhang WG, Yuan JB, Li YG, Yang SL, Yang WL: **Simultaneous quantification of polymethoxylated flavones and coumarins in Fructus aurantii and Fructus aurantii immaturus using HPLC-ESI-MS/MS.** *J Pharmaceut Biomed* 2012, **59**: 90-95.

14. Hirata T, Fujii M, Akita K, Yanaka N, Ogawa K, Kuroyanagi M, Hongo D: **Identification and physiological evaluation of the components from Citrus fruits as potential drugs for anti-corpulence and anticancer.** *Bioorgan Med Chem* 2009 **17**: 25-28.

15. Dugrand A, Olry A, Duval T, Hehn A, Froelicher Y, Bourgaud F: **Coumarin and Furanocoumarin Quantitation in Citrus Peel via Ultraperformance Liquid Chromatography Coupled with Mass Spectrometry (UPLC-MS).** *J Agr Food Chem* 2013, **61**: 10677-10684.

16. Mercolini L, Mandrioli R, Ferranti A, Sorella V, Protti M, Epifano F, Curini M, Raggi MA: **Quantitative evaluation of auraptene and umbelliferone, chemopreventive coumarins in citrus fruits by HPLC-UV-FL-MS.** *J Agric Food Chem* 2013, **61**: 1694-1701.

17. Thongthoom T, Songsiang U, Phaosiri C, Yenjai C: **Biological activity of chemical constituents from Clausena harmandiana.** *Arch Pharm Res* 2010, **33**: 675-680.

18. Patil JR, Jayaprakasha GK, Kim J, Murthy KN, Chetti MB, Nam SY, Patil BS: **5-Geranyloxy-7-methoxycoumarin inhibits colon cancer (SW480) cells growth by inducing apoptosis.** *Planta Med* 2013, **79**: 219-226.

19. Gorgus E, Lohr C, Raquet N, Guth S, Schrenk D: **Limettin and furocoumarins in beverages containing citrus juices or extracts.** *Food Chem Toxicol* 2010, **48**: 93-98.

20. Pellati F, Benvenuti S: **Fast high-performance liquid chromatography analysis of phenethylamine alkaloids in Citrus natural products on a pentafluorophenylpropyl stationary phase.** *J Chromatogr A* 2007, **1165**: 58-66.

21. Davide Barreca EB, Corrado Caristi, Ugo Leuzzi, Giuseppe Gattuso: **Distribution of C- and O-glycosyl flavonoids, (3-hydroxy-3-methylglutaryl) glycosyl flavanones and furocoumarins in Citrus aurantium L. juice.** *Food Chem* 2011, **124**: 576–582.

22. Patil JR, Chidambara Murthy K, Jayaprakasha G, Chetti MB, Patil BS: **Bioactive compounds from Mexican lime (Citrus aurantifolia) juice induce apoptosis in human pancreatic cells.** *J Agric Food Chem* 2009, **57**: 10933-10942.

23. Breksa III AP, Kahn T, Zukas AA, Hidalgo MB, Yuen ML: **Limonoid content of sour orange varieties.** *J Sci Food Agric* 2011, **91**: 1789-1794.

24. Jayaprakasha GK, Dandekar DV, Tichy SE, Patil BS: **Simultaneous separation and identification of limonoids from citrus using liquid chromatography-collision-induced dissociation mass spectra.** *J Sep Sci* 2011, **34**: 2-10.

25. Kim J, Jayaprakasha GK, Patil BS: **Limonoids and their anti-proliferative and anti-aromatase properties in human breast cancer cells.** *Food Funct* 2013, **4**: 258-265.

26. Liao J, Xu T, Liu YH, Wang SZ: **A new limonoid from the seeds of Citrus reticulata Blanco.** *Nat*

Prod Res 2012, **26**: 756-761.

27. Mahmoud MF, Hamdan DI, Wink M, El-Shazly AM: **Hepatoprotective effect of limonin, a natural limonoid from the seed of Citrus aurantium var. bigaradia, on D-galactosamine-induced liver injury in rats.** *N-S Arch Pharmacol* 2014, **387**: 251-261.

28. Breksa III AP, Hidalgo MB, Yuen ML: **Liquid chromatography-electrospray ionisation mass spectrometry method for the rapid identification of citrus limonoid glucosides in citrus juices and extracts.** ***Food chemistry*** 2009, **117**: 739-744.

29. Bermejo A, Llosa MJ, Cano A: **Analysis of bioactive compounds in seven citrus cultivars.** *Food Sci Technol Int* 2011, **17**: 55-62.

30. Carmona L, Zacarías L, Rodrigo MJ: **Stimulation of coloration and carotenoid biosynthesis during postharvest storage of 'Navelina'orange fruit at 12℃.** *Postharvest Biol Tec* 2012, **74**: 108-117.

31. Poiroux-Gonord F, Fanciullino AL, Bert L, Urban L: **Effect of fruit load on maturity and carotenoid content of clementine (Citrus clementina Hort. ex Tan.) fruits.** *J Sci Food Agric* 2012, **92**: 2076-2083.

32. Rodrigo MJ, Marcos JF, Zacarias L: **Biochemical and molecular analysis of carotenoid biosynthesis in flavedo of orange (Citrus sinensis L.) during fruit development and maturation.** *J Agric Food Chem* 2004, **52**: 6724-6731.

33. Cheong MW, Chong ZS, Liu SQ, Zhou W, Curran P, Bin Y: **Characterisation of calamansi (Citrus microcarpa). Part I: volatiles, aromatic profiles and phenolic acids in the peel.** *Food Chem* 2012, **134**: 686-695.

34. Ma YQ, Ye XQ, Fang ZX, Chen JC, Xu GH, Liu DH: **Phenolic compounds and antioxidant**

activity of extracts from ultrasonic treatment of Satsuma Mandarin (Citrus unshiu Marc.) peels. *J Agric Food Chem* 2008, **56**: 5682-5690.

35. Manthey JA, Grohmann K: **Phenols in citrus peel byproducts. Concentrations of hydroxycinnamates and polymethoxylated flavones in citrus peel molasses.** *J Agric Food Chem* 2001, **49**: 3268-3273.

36. Xu G, Ye X, Liu D, Ma Y, Chen J: **Composition and distribution of phenolic acids in Ponkan (Citrus poonensis Hort. ex Tanaka) and Huyou (Citrus paradisi Macf. Changshanhuyou) during maturity.** *J Food Compos Anal* 2008, **21**: 382-389.

37 Moulehi I, Bourgou S, Ourghemmi I, Tounsi MS: **Variety and ripening impact on phenolic composition and antioxidant activity of mandarin (Citrus reticulate Blanco) and bitter orange (Citrus aurantium L.) seeds extracts.** *Ind Crop Prod* 2012, **39:** 74-80.

38. Chen ML, Yang DJ, Liu SC: **Effects of drying temperature on the flavonoid, phenolic acid and antioxidative capacities of the methanol extract of citrus fruit (Citrus sinensis (L.) Osbeck) peels.** *Int J Food Sci Tech* 2011, **46**: 1179-1185.

39. Hayat K, Hussain S, Abbas S, Farooq U, Ding B, Xia S, Jia C, Zhang X, Xia W: **Optimized microwave-assisted extraction of phenolic acids from citrus mandarin peels and evaluation of antioxidant activity in vitro.** *Sep Purif Technol* 2009, **70**: 63-70.

40. Jabri Karoui I, Marzouk B: **Characterization of bioactive compounds in Tunisian bitter orange (Citrus aurantium L.) peel and juice and determination of their antioxidant activities.** *Biomed Res Int* 2013, **2013:** 345415.

41. Arbo MD, Larentis ER, Linck VM, Aboy AL, Pimentel AL, Henriques AT, Dallegrave E, Garcia SC, Leal MB, Limberger RP: **Concentrations of p-synephrine in fruits and leaves of Citrus**

species (Rutaceae) and the acute toxicity testing of Citrus aurantium extract and p-synephrine. *Food Chem Toxicol* 2008, **46**: 2770-2775.

42. Abad-Garcia B, Berrueta LA, Garmon-Lobato S, Urkaregi A, Gallo B, Vicente F: **Chemometric characterization of fruit juices from Spanish cultivars according to their phenolic compound contents: I. Citrus fruits.** *J Agric Food Chem* 2012, **60**: 3635-3644.
