# Supplementary material for: Citrus fruits as a treasure trove of active natural metabolites that potentially provide benefits for human health
Source: Chem Cent J. 2015 Dec 24;9:68. doi: 10.1186/s13065-015-0145-9 (PMC4690266; doi:10.1186/s13065-015-0145-9)
Supplement: Supplementary file 3 — 10.1186/s13065-015-0145-9 Volatile compounds isolated from Citrus species. The table summarized citrus-derived volatile compounds from common Citrus Species including C. Aurantium, C. Aurantifolia, C. Medica, C. Limon, C. Bergamia, Citrus reticulata, C. Kinokuni, C. Unshiu, C. Clementina, C. Sinensis, C. Clementine × C. Tangerine, C. grandis × C. Grandis, C. Paradisi, C. Nobilis and C. depressa. [file 13065_2015_145_MOESM3_ESM.pdf]

Additional file 3 volatile compounds isolated from *Citrus* species.

compounds	source	ref.
α-pinene	CGG[d], CCL[a], CCL[d], CCLT[d], CSI[a], CSI[c], CSI[d], CAT[a], CAT[d], CAT[e], CU[a], CLI[a], CLI[c], CR[a], CR[c], CK[a], CME[c], CAF[e], CB[e], CME[c], CP[a], CP[c], CN[a], CD[a]	[1-15]
β-pinene	CAT[a], CAT[e], CCL[a], CCL[d], CU[a], CSI[a], CSI[c], CSI[d], CGG[d], CCLT[d], CLI[a], CLI[c], CR[a], CR[c], CME[c], CAF[e], CB[e], CME[c], CP[a], CP[c], CN[a], CD[a]	[1-4,6-15]
sabinene	CAT[a], CLI[c], CR[c], CSI[c], CME[c], CAF[e], CAT[e], CB[e], CME[c], CD[a], CN[a], CP[a], CLI[a], CR[a], CSI[a]	[2-4,6,8,10,11,13-15]
myrcene	CAT[a], CAT[d], CCL[a], CCL[d], CU[a], CSI[a], CSI[c], CSI[d], CGG[d], CCLT[d], CLI[c], CR[c], CK[a], CME[c], CAF[e], CAT[e], CB[e], CME[c], CP[c], CN[a], CD[a], CP[a], CLI[a], CR[a]	[1-6,8-15]
α-terpinene	CAT[a], CAT[e], CGG[d], CCL[d], CCLT[d], CSI[d], CLI[a], CLI[c], CME[c], CAF[e], CME[c], CD[a], CP[a], CR[a], CSI[a]	[1,3,4,6-8,10,14,15]
γ-terpinene	CGG[d], CCL[d], CCLT[d], CSI[d], CLI[a], CLI[c], CK[a], CU[a], CME[c], CAF[e], CAT[e], CB[e], CME[c], CD[a], CN[a], CP[a], CR[a], CSI[a]	[1,4-8,10,11,13,14,15]
p-cimene	CME[c]	[6]
1,4-cineole	CCL[d], CSI[d]	[1]
1,8-cineole	CAT[a], CAT[d], CLI[c], CGG[d], CCL[d], CCLT[d], CSI[d]	[1,3,4]
1,8-cineol	CD[a]	[14]
E-*β*-ocimene	CAT[a], CLI[c], CME[c], CAF[e], CB[e], CME[c], CN[a], CP[a], CLI[a], CR[a], CSI[a]	[3,4,6,8,10,15]
cis-β-ocimene	CAT[a]	[2]
(*Z*)-*β*-ocimene	CAF[e], CAT[e], CME[c], CN[a], CP[a], CLI[a], CR[a], CSI[a]	[8,10,15]
(Z)-ocimene	CGG[d]	[1]
terpinolene	CAT[a], CAT[e], CGG[d], CCL[d], CCLT[d], CSI[c], CSI[d], CLI[a], CLI[c], CME[c], CAF[e], CB[e], CD[a], CN[a], CP[a], CR[a], CSI[a]	[1,3,4,6-8,10,11,13-15]
cis-linalool oxide	CAT[a], CAT[d], CAT[e], CAF[e], CB[e]	[3,8]
trans-linalool oxide	CAT[a], CAT[d]	[3]

(Z)-linalool oxide	CGG[d], CCLT[d], CN[a]	[1,15]
(E)-linalool oxide	CGG[d], CN[a]	[1,15]
linalool	CAT[a], CAT[e], CLI[c], CR[c], CME[c], CAF[e], CB[e], CCL[a], CCL[d], CU[a], CSI[a], CSI[c], CSI[d], CGG[d], CCLT[d], CN[a], CD[a], CP[a], CLI[a], CR[a]	[1-4,6,8-10,12,14,15]
linalyl acetate	CAT[a], CAT[e], CGG[d], CCL[d], CCLT[d], CSI[d], CAF[e], CB[e]	[1-3,8]
neryl acetate	CAT[a], CAT[e], CAT[d], CB[e], CGG[d], CCL[d], CCLT[d], CSI[d], CME[c], CLI[a], CLI[c], CAF[e], CN[a], CP[a], CR[a], CSI[a]	[1-3,6-8,10,11,12,15]
geranyl acetate	CAT[a], CAT[e], CGG[d], CCL[d], CCLT[d], CSI[d], CME[c], CAF[e], CB[e], CLI[c], CN[a], CP[a], CLI[a], CR[a], CSI[a]	[1,3,6,8,10,11,12,15]
bornyl acetate	CGG[d], CCL[d], CCLT[d], CSI[d], CAT[a], CAF[e], CD[a]	[1,3,8,14]
propyl acetate	CCLT[d]	[1]
farnesyl acetate	CAT[a]	[3]
terpinene-4-ol	CAT[a]	[3]
caryophyllene	CAT[a], CAT[e], CGG[d], CSI[c], CSI[d], CLI[c], CME[c], CAF[e], CB[e], CD[a], CN[a], CP[a], CLI[a], CR[a], CSI[a]	[1,3,4,6,8,10,11,13-15]
β-elemene	CSI[c], CME[c], CN[a], CP[a], CLI[a], CR[a], CSI[a]	[4,6,10,15]
γ-elemene	CAT[a]	[3]
δ-elemene	CME[c], CN[a], CP[a], CSI[a]	[10, 15]
neral	CAT[a], CAT[e], CGG[d], CCL[d], CCLT[d], CSI[d], CME[c], CLI[a], CAF[e], CLI[c], CN[a], CP[a], CR[a], CSI[a]	[1,3,6-8,10-13, 15]
nerol	CAT[a], CAT[e], CGG[d], CCL[d], CCLT[d], CSI[d], CME[c], CLI[a], CAF[e], CLI[c], CN[a], CD[a]	[1,3,6-8,11,12,14,15]
α-farnesene	CLI[c], CN[a], CR[a],	[13,15]
β-farnesene	CAT[a], CAT[e], CSI[d], CLI[c], CME[c], CAF[e], CB[e], CLI[a]	[1,3,4,6,8,10,15]

α-terpineol	CAT[a], CAT[d], CAT[e], CLI[c], CME[c], CAF[e], CB [e]	[2,3,4,6,8,10]
δ-cadinene	CAT[a], CSI[c], CME[c], CD[a], CN[a], CR[a], CSI[a]	[3,4,6,14,15]
γ-cadinene	CME[c], CAF[e], CAT[e]	[6,8]
geraniol	CAT[a], CME[c], CLI[a], CLI[c], CGG[d], CCL[d], CCLT[d], CSI[d], CN[a], CP[a], CLI[a], CR[a], CSI[a]	[1,3,6,7,10,11,12,15]
nerolidol	CAT[a], CAT[e], CN[a], CP[a], CSI[a]	[3,8,15]
thujene	CME[c], CAF[e], CAT[d], CAT[e], CB[e], CLI[a], CLI[c], CME[c], CD[a], CN[a], CP[a], CR[a], CSI[a]	[3,4,6,8,10,13,14,15]
tricyclen	CAT[d]	[3]
α-phellandrene	CAT[d], CAT[e], CSI[a], CSI[d], CGG[d], CCL[d], CCLT[d], CME[c], CAF[e], CME[c], CN[a], CP[a], CLI[a], CR[a]	[1,3,6-8,10,15]
β-phellandrene	CLI[c], CAT[c], CAT[e], CAF[e], CD[a], CN[a], CP[a], CLI[a], CR[a], CSI[a]	[4,8,14,15]
Oxyde decaryophyllene	CAT[d]	[3]
Octanal	CAT[a], CAT[c], CGG[d], CCL[d], CCLT[d], CSI[d], CLI[c], CR[c], CME[c]	[1,2,4,10]
limonene	CAT[a], CAT[d], CAF[e], CCL[a], CCL[d], CU[a], CSI[a], CSI[c], CSI[d], CGG[d], CCLT[d], CLI[a], CLI[c], CR[c], CK[a], CU[a], CME[c], CB[e], CME[c], CP[c], CN[a], CD[a], CP[a], CR[a]	[1-10,12-15]
α-terpineol	CAT[a], CCL[a], CCL[d], CU[a], CSI[a], CSI[d], CGG[d], CCLT[d], CLI[c], CD[a], CN[a], CP[a], CLI[a], CR[a]	[1,2,4,9,11,14,15]
decanal	CAT[a], CGG[d], CCL[d], CCLT[d], CSI[d], CR[c], CSI[c], CME[c], CN[a], CD[a]	[1,2,4,9,10,12,14]
n-decanal	CME[c], CAF[e], CAT[e], CB[e]	[6,8]
citronellal	CCL[a], CCL[d], CU[a], CSI[a], CSI[c], CLI[c], CR[c], CME[c], CAF[e], CAT[e], CN[a], CLI[a], CR[a]	[1,4,6,8-10,12,15]
2-carene	CGG[d], CCLT[d], CSI[d]	[1]
3-carene	CGG[d], CCL[d], CCLT[d], CSI[d], CAF[e], CAT[e], CME[c]	[1,8,10]
4-carene	CD[a]	[14]
2,4-nonadienal	CGG[d]	[1]

2,3-pentanedione	CGG[d], CCL[d], CCLT[d], CSI[d]	[1]
2,4-decadienal	CGG[d], CCL[d], CCLT[d], CSI[d], CN[a]	[1,12]
p-cymene	CGG[d], CLI[c], CR[c], CSI[c], CAF[e], CAT[e], CD[a]	[1,4,8,13,14]
nootkatone	CGG[d], CCLT[d], CSI[c], CSI[d], CLI[c], CME[c]	[1,4,10]
α-humulene	CGG[d], CCL[d], CCLT[d], CSI[d], CME[c], CAF[e], CAT[e], CB[e], CLI[c]	[1,6,8,10,13]
1-pentanol	CGG[d], CCL[d], CCLT[d], CSI[d]	[1]
1-hexanol	CGG[d], CCL[d], CCLT[d], CSI[d]	[1]
methyl hexanoate	CGG[d], CCLT[d], CSI[d]	[1]
ethyl hexanoate	CGG[d], CCL[d], CCLT[d], CSI[d]	[1]
pseudocumene	CGG[d], CCL[d], CCLT[d], CSI[d]	[1]
6-methyl-5-hepten-2-one	CGG[d], CCL[d], CCLT[d]	[1]
heptanal	CGG[d], CCL[d], CCLT[d], CSI[d]	[1]
nonanal	CGG[d], CCL[d], CCLT[d], CSI[d], CAF[e], CAT[e], CB[e], CME[c], CN[a], CD[a]	[1,8,10,12,14]
1-nonanol	CGG[d], CCL[d], CCLT[d], CSI[d], CN[a]	[1,12]
n-nonanal	CLI[c], CSI[c], CME[c]	[4,6]
2-octenal	CGG[d], CCL[d], CCLT[d], CSI[d]	[1]
2-nonenal	CGG[d], CCL[d], CCLT[d], CSI[d], CN[a]	[1,12]
2-heptenal	CGG[d], CCL[d], CCLT[d], CSI[d]	[1]
1-octen-3-one	CGG[d], CCL[d], CCLT[d], CSI[d]	[1]
1-octen-3-ol	CGG[d], CCL[d], CCLT[d], CSI[d]	[1]
hexanal	CGG[d], CCL[d], CCLT[d], CSI[d]	[1]
pentanal	CGG[d], CCL[d], CCLT[d], CSI[d]	[1]
2-pentylfuran	CGG[d], CCL[d], CCLT[d], CSI[d]	[1]
1-heptanol	CGG[d], CCL[d], CCLT[d], CSI[d]	[1]
α-copaene	CGG[d], CCL[d], CCLT[d], CSI[d], CD[a], CN[a], , CR[a], CSI[a]	[1,14,15]
β-copaene	CR[c], CSI[c]	[4]
valencene	CGG[d], CCL[d], CCLT[d], CSI[d], CLI[c], CR[c], CME[c], CLI[a], CR[a]	[1,4,10,15]
ethyl heptanoate	CGG[d], CCL[d], CCLT[d], CSI[d]	[1]
2-ethylfuran	CGG[d], CCL[d], CCLT[d], CSI[d]	[1]
2-methylfuran	CGG[d], CCL[d], CCLT[d], CSI[d]	[1]
3- methylfuran	CGG[d], CCL[d], CCLT[d], CSI[d]	[1]
2-pentenal	CGG[d], CCL[d], CCLT[d], CSI[d]	[1]
1-penten-3-one	CGG[d], CCL[d], CCLT[d], CSI[d]	[1]
ethyl propanoate	CGG[d], CCL[d], CCLT[d], CSI[d]	[1]
ethyl 2-methylbutanoate	CGG[d], CCL[d], CCLT[d], CSI[d]	[1]
3-pentanone	CGG[d], CCL[d], CCLT[d], CSI[d]	[1]
1-penten-3-ol	CGG[d], CCL[d], CCLT[d], CSI[d]	[1]
(Z)-3-hexenal	CGG[d], CCL[d], CCLT[d], CSI[d]	[1]
(E)-2-hexenal	CGG[d], CCL[d], CCLT[d], CSI[d]	[1]
geranylacetone	CGG[d], CCL[d], CCLT[d], CSI[d]	[1]

β-cyclocitral	CGG[d], CCL[d], CCLT[d], CSI[d]	[1]
β-ionone	CGG[d], CCL[d], CCLT[d], CSI[d]	[1]
hexyl acetate	CGG[d], CCL[d], CCLT[d], CSI[d]	[1]
ethyl nonanoate	CGG[d], CCL[d], CCLT[d], CSI[d]	[1]
methyl nonanoate	CGG[d], CCL[d], CCLT[d], CSI[d]	[1]
(Z)-3-hexen-1-ol	CGG[d], CCL[d], CCLT[d], CSI[d]	[1]
ethyl octanoate	CGG[d], CCL[d], CCLT[d], CSI[d]	[1]
methyl octanoate	CGG[d], CCL[d], CCLT[d], CSI[d]	[1]
gerania	CGG[d], CCL[d], CCLT[d], CSI[d]	[1]
heptyl acetate	CGG[d], CCL[d], CCLT[d], CSI[d]	[1]
ethyl decanoate	CGG[d], CCL[d], CCLT[d], CSI[d]	[1]
methyl decanoate	CGG[d], CCL[d], CCLT[d], CSI[d]	[1]
undecanal	CGG[d], CCL[d], CCLT[d], CSI[d], CME[c], CN[a]	[1,10,12]
(E)-2-hexen-1-ol	CCL[d], CSI[d]	[1]
1-decanol	CCL[d], CCLT[d], CSI[d], CN[a]	[1,12]
1-octanol	CGG[d], CCL[d], CCLT[d], CSI[d]	[1]
n-octanol	CSI[c]	[4]
citronellol	CLI[c], CGG[d], CCL[d], CCLT[d], CSI[d], CME[c], CN[a], CP[a], CLI[a], CR[a], CSI[a]	[1,4,10,12,15]
ethyl acetate	CGG[d], CCL[d], CCLT[d], CSI[d]	[1]
carveol	CGG[d], CCL[d], CCLT[d], CSI[d], CLI[c], CR[c], CSI[c], CN[a], CD[a]	[1,4,12,14]
carvone	CGG[d], CCL[d], CCLT[d], CSI[d], CLI[c], CR[c], CSI[c], CN[a], CD[a]	[1,4,12,14]
ethanol	CGG[d], CCL[d], CCLT[d], CSI[d]	[1]
acetaldehyde	CGG[d], CCL[d], CCLT[d], CSI[d]	[1]
dodecanal	CGG[d], CCL[d], CCLT[d], CSI[d], CME[c], CN[a]	[1,10,12]
limonene oxide	CGG[d], CCL[d], CCLT[d], CSI[d], CLI[c], CSI[c], CME[c], CN[a], CP[a], CLI[a], CR[a], CSI[a]	[1,4,10,15]
camphene	CGG[d], CCL[d], CCLT[d], CSI[d], CME[c], CAF[e], CAT[e], CB[e], CME[c], CD[a], CN[a], CP[a], CLI[a], CR[a], CSI[a]	[1,6,8,10,14,15]
camphor	CME[c], CN[a], CLI[a]	[10,12,15]
terpinen-4-ol	CGG[d], CCL[d], CCLT[d], CSI[d], CME[c], CAF[e], CAT[e], CB[e], CLI[c], CD[a]	[1,6,8,10,11,14]
perillaldehyde	CGG[d], CCL[d], CCLT[d], CSI[d]	[1]
carvyl acetate	CGG[d], CCL[d], CCLT[d], CSI[d]	[1]
citronellyl acetate	CGG[d], CCL[d], CCLT[d], CSI[d], CME[c], CLI[a],	[1,6,10,15]

	CSI[a]	
styrene	CGG[d], CCL[d], CCLT[d], CSI[d]	[1]
nonyl acetate	CGG[d], CCL[d], CCLT[d], CSI[d]	[1]
ethyl butanoate	CGG[d], CCL[d], CCLT[d], CSI[d]	[1]
decyl acetate	CGG[d], CCL[d], CCLT[d], CSI[d]	[1]
terpinyl acetate	CCL[d], CCLT[d], CSI[d], CD[a]	[1,14]
octyl acetate	CGG[d], CCL[d], CCLT[d], CSI[d], CME[c]	[1,10]
3-methylbutanal	CCL[d]	[1]
γ-dodecalactone	CGG[d]	[1]
iso-sylvestrene	CSI[c]	[4]
sabinene hydrate	CLI[c], CME[c], CD[a], CN[a], CP[a], CLI[a], CR[a], CSI[a]	[4,6,10,14,15]
trans-p-mentha-2,8-dienol	CR[c], CSI[c]	[4]
cis-p-mentha-2,8-dienol	CLI[c], CR[c], CSI[c]	[4]
terpineol-4-ol	CLI[c]	[4]
cis-pinocarveol	CR[c], CSI[c]	[4]
cryptone	CR[c], CSI[c]	[4]
p-mentha-1,8-dien-7-ol	CR[c]	[4]
cis-p-mentha-1(7),8-dien-2-ol	CLI[c], CSI[c]	[4]
p-mentha-1(7),8(10)-dien-9-ol	CR[a]	[15]
geranial	CLI[a], CLI[c], CSI[c], CME[c], CAF[e], CAT[e], CB[e], CN[a], CP[a], CR[a], CSI[a]	[4,6-8,10-13, 15]
p-mentha-1,8-dien-3-one	CR[c], CSI[c], CN[a]	[4,15]
perilla aldehyde	CLI[c], CR[c], CSI[c], CME[c], CN[a], CD[a]	[4,6,10,12,14]
thymol	CLI[c], CR[c], CSI[c], CD[a]	[4,14]
isothymol	CD[a]	[14]
carvacrol	CLI[c], CR[c], CSI[c], CME[c]	[4,10]
perilla alcohol	CR[c], CSI[c]	[4]
patchenol	CR[c], CSI[c]	[4]
β-cubenene	CR[c], CSI[c]	[4]
sativene	CSI[c]	[4]
(Z)-trimenal	CR[c]	[4]
α-bergamotene	CME[c], CAF[e], CAT[e], CB[e], CLI[c], CLI[a]	[4,6,8,10,11,15]
cis-thujopsene	CLI[c]	[4]

β-santalene	CLI[c], CME[c]	[4,10]
germacrene D	CLI[c], CME[c], CAF[e], CAT[e], CD[a], CN[a], CP[a], CR[a], CSI[a]	[4,6,8,14,15]
germacrene B	CP[a]	[15]
β-selinene	CLI[c]	[4]
7-epi-α-selinene	CSI[c]	[4]
bicyclogermacrene	CLI[c], CME[c]	[4,10]
α-muurolene	SCI[c], CD[a], CN[a], CR[a]	[4,14,15]
γ -muurolene	CD[a]	[14]
β-bisabolene	CLI[c], CME[c], CAF[e], CAT[e], CB[e], CN[a], CLI[a]	[4,6,8,10,15]
(Z)-a-bisabolene	CME[c], CLI[a]	[10,15]
γ-bisabolene	CME[c]	[10]
spathulenol	CLI[c], CSI[c]	[4]
caryophyllene oxide	CLI[c], CSI[c], CAF[e], CAT[e], CB[e]	[4,8]
a-bisabolool	CLI[c]	[4]
β-sinensal	CSI[c]	[4]
(3E,5Z)-undeca-1,3,5-triene	CK[a], CU[a]	[5]
(3E,5Z.8Z)-undeca-1,3,5,8-tetraene	CK[a], CU[a]	[5]
2,3-dihydro-3,5-dihydroxy-6-methyl-4H-Pyran-4-one	CME[c]	[6]
2,3-dihydrobenzofuran	CME[c]	[6]

α-cubebene	CD[a]	[14]
β-cubebene	CME[c], CN[a], CR[a],	[6,15]
tetradecanal	CME[c], CAF[e], CAT[e]	[6,8,10]
α-bisabolol	CAF[e], CAT[e], CME[c], CLI[a]	[8,10,15]
β-bisabolol	CME[c]	[6]
eicosane	CME[c], CAF[e], CAT [e]	[6, 8]
docosane	CME[c]	[6]
citropten	CME[c]	[6]
oxypeucedanin	CME[c]	[6]
alloocimene	CAT[e]	[8]
fenchyl acetate	CAF[e], CAT[e]	[8]
bicyclosesquiphellandren e	CAF[e], CAT[e], CD[a]	[8,14]
osthol	CAT[e]	[8]
manoyl oxide	CAF[e], CAT[e]	[8]
heneicosane	CAF[e], CAT[e]	[8]
tricosane	CAF[e], CAT[e]	[8]
pentacosane	CAF[e], CAT[e]	[8]
nonacosane	CAF[e], CAT[e]	[8]
decane	CME[c]	[10]
borneol	CME[c], CLI[a]	[10, 15]
p-cymen-8-ol	CME[c]	[10]
dihydrolinalyl acetate	CME[c]	[10]
norbornanol	CME[c]	[10]
campherenol	CME[c]	[10]
hexadecanal	CME[c]	[10]
linanool	CSI[c], CP[c]	[11]
1-phenylethyl mercaptan	CN[a]	[12]
(*E*,*Z*)-2,6- nonadien-1-ol	CN[a]	[12]
2-methoxy-3-(2-methylpr opyl) pyrazine	CN[a]	[12]
4-terpineol	CN[a], CP[a], CLI[a], CR[a], CSI[a]	[12,15]
(*E*)-2-decenal	CN[a], CD[a]	[12,14]
(*Z*)-5-dodecenal	CN[a]	[12]
(E,Z)-2,6-dodecadienal	CN[a]	[12]

(E)-2-dodecenal	CN[a]	[12]
α-bergamottene	CLI[a]	[13]
elemol	CD[a], CN[a], CR[a], CSI[a]	[14,15]
γ-eudesmol	CD[a]	[14]
cosmene	CP[a]	[15]
(Z)-p-menth-2-en-1-ol	CN[a], CP[a], CLI[a]	[15]
(E)-piperitol	CSI[a]	[15]
8-hydroxylinalool	CN[a], CLI[a]	[15]
perillal	CN[a], CLI[a], CR[a], CSI[a]	[15]
piperitone	CN[a], CLI[a]	[15]
methyl geranate	CN[a], CLI[a]	[15]
geranyl isobutyrate	CLI[a]	[15]
α-guaiene	CN[a]	[15]
elixene	CN[a], CLI[a], CR[a], CSI[a]	[15]
cadalene	CN[a], CP[a], CR[a], CSI[a]	[15]
germacrene D-4-ol	CN[a], CLI[a], CR[a], CSI[a]	[15]

a: peel; c: pressed oil; d: juice; e: whole fruit; CAT: *C. aurantium;* CAF: *C. aurantifolia*; CME: *C. medica*; CLI: *C. limon*; CB: *C. bergamia; CR: Citrus reticulata*; CK: *C. kinokuni*; CU: *C. unshiu*; CCL: *C. clementina*; CSI: *C. sinensis*; CCLT: *C. Clementine* × *C. tangerine*; CGG: *C. grandis* × *C. grandis*; CP: *C. paradisi;* CN: *C. nobilis;* CD: *C. depressa.*

1. M. Carmen GM, Jose´ LR, M. Carmen A, Abelardo G, Antonio G: **Comparative analysis of the volatile fraction of fruit juice from different Citrus species.** *Plos One* 2011, **6**: 1-11.

2. Eirini S, Paschalina C, Kortessa DT, Ioannis T: **Volatile constituents and antioxidant activity of peel, flowers and leaf oils of *Citrus aurantium* L. growing in Greece.** *Molecules* 2013, **18**: 10639-10647.

3. Iness JK, Brahim M: **Characterization of bioactive compounds in Tunisian bitter orange (*Citrus aurantium* L.) peel and juice and determination of their antioxidant activities.** *Biomed Res Int* 2013, **2013**:1-12.

4. Laura E, María S, Susana L, Pilar C, Diego G, Rafael P: **Chemical composition of commercial citrus fruit essential oils and evaluation of their antimicrobial activity acting alone or in combined processes.** *Food Control* 2011, **22**: 896-902.

5. Norio M, Akira F, Kikue K: **Aroma character impact compounds in kinokuni mandarin orange (Citrus kinokuni) compared with satsuma mandarin orange (Citrus unshiu).** *Biosci Biotechnol Biochem* 2010, **74**: 835-842.

6. Federica M, Rosa T, Marco B, Bruno de C, Monica RL, Filomena C, Giancarlo AS , Roberta M, Ruggero B, Francesco M: **Chemical composition and bioactivity of Citrus medica L. cv. Diamante essential oil obtained by hydrodistillation, cold-pressing and supercritical carbon dioxide extraction.** *Nat Prodt Res* 2011, **25**: 789-799.

7. Antonios M, Dimitrios P, Athanasios K, George K, Athanasios G, Moschos GP: **Citrus essential oils and four enantiomeric pinenes against Culex pipiens (Diptera: Culicidae).** *Parasitol Res* 2009, **105:**769–773.

8. Rosa T, Monica RL, Marco B, Federica M, Vincenzo M, Carmela C, Francesco M: **Comparative study on the antioxidant capacity and cholinesterase inhibitory activity of Citrus aurantifolia Swingle, C. aurantium L., and C. bergamia Risso and Poit. peel essential oils.** *J Food Sci* 2012, **71**: 40-46.

9. A Bermejo, MJ Llosa′, A Cano: **Analysis of bioactive compounds in seven Citrus cultivars.** *Food Sci Tech Int* 2011, **17**: 55-58.

10. Bartolo G, Alessia F, Paola D, Rosaria C, Luigi M: **Essential oil composition of Citrus medica L. Cv. Diamante (Diamante citron) determined after using different extraction methods.** *J Sep Sci* 2009, **32**: 99-108.

11. Athanassios G, Dimitrios PP, Athanasios K, George K, Moschos GP, Nickolaos E, Antonios M: **Evaluation of bioefficacy of three Citrus essential oils against the dengue vector Aedes albopictus (Diptera: Culicidae) in correlation to their components enantiomeric distribution.** *Parasitol Res* 2012, **111**: 2253–2263.

12. Jorry D, Stefan K, Praveena S, Martin JL, Philip C: **Evaluation of aroma-active compounds in pontianak orange peel oil (*Citrus nobilis* Lour. Var. *microcarpa* Hassk.) by gas chromatography-olfactometry, aroma reconstitution, and omission test.** *J. Agric. Food Chem.* 2009, **57:** 239–244.

13. L Valgimigli, S Gabbanini, E Berlini, E Lucchi, C Beltramini, YL Bertarelli: **Lemon (Citrus limon, Burm.f.) essential oil enhances the trans-epidermal release of lipid- (A, E) and water- (B6, C) soluble vitamins from topical emulsions in reconstructed human epidermis.** *Int J Cosmetic Sci* 2012, **34**: 347–356.

14. Yonathan A, Ikuko T, Sayuri I, Hidekazu S, Masayoshi S, Kensaku T, Koji W: **Volatile aroma components and antioxidant activities of the flavedo peel extract of unripe shiikuwasha (*Citrus depressa* Hayata).** *J Food Sci* 2012, 77: c469-c475.

15. Cuihua L, Yunjiang C, Hongyan Z, Xiuxin D, Feng C, Juan X: **Volatile constituents of wild Citrus mangshanyegan (Citrus nobilis Lauriro) Peel Oil.** *J Agric Food Chem* 2012, **60**: 2617−2628.
